# Detection Games under Fully Active Adversaries

**DOI:** 10.3390/e21010023

**Published:** 2018-12-29

**Authors:** Benedetta Tondi, Neri Merhav, Mauro Barni

**Affiliations:** 1 Department of Information Engineering and Mathematical Sciences, University of Siena, 53100 Siena, Italy; 2 The Andrew and Erna Viterbi Faculty of Electrical Engineering—Israel Institute of Technology Technion City, Haifa 3200003, Israel

**Keywords:** adversarial signal processing, binary hypothesis testing, statistical detection theory, game theory, the method of types

## Abstract

We study a binary hypothesis testing problem in which a defender must decide whether a test sequence has been drawn from a given memoryless source $P_{0}$, while an attacker strives to impede the correct detection. With respect to previous works, the adversarial setup addressed in this paper considers an attacker who is active under both hypotheses, namely, a fully active attacker, as opposed to a partially active attacker who is active under one hypothesis only. In the fully active setup, the attacker distorts sequences drawn both from $P_{0}$ and from an alternative memoryless source $P_{1}$, up to a certain distortion level, which is possibly different under the two hypotheses, to maximize the confusion in distinguishing between the two sources, i.e., to induce both false positive and false negative errors at the detector, also referred to as the defender. We model the defender–attacker interaction as a game and study two versions of this game, the Neyman–Pearson game and the Bayesian game. Our main result is in the characterization of an attack strategy that is asymptotically both dominant (i.e., optimal no matter what the defender’s strategy is) and universal, i.e., independent of $P_{0}$ and $P_{1}$. From the analysis of the equilibrium payoff, we also derive the best achievable performance of the defender, by relaxing the requirement on the exponential decay rate of the false positive error probability in the Neyman–Pearson setup and the tradeoff between the error exponents in the Bayesian setup. Such analysis permits characterizing the conditions for the distinguishability of the two sources given the distortion levels.

## 1. Introduction

Signal processing techniques are routinely applied in the great majority of security-oriented applications. In particular, the detection problem plays a fundamental role in many security-related scenarios, including secure detection and classification, target detection in radar-systems, intrusion detection, biometric-based verification, one-bit watermarking, steganalysis, spam-filtering, and multimedia forensics (see [1]). A unifying characteristic of all these fields is the presence of an adversary explicitly aiming at hindering the detection task, with the consequent necessity of adopting proper models that take into account the interplay between the system designer and the adversary. Specifically, the adopted models should be able to deal, on the one hand, with the uncertainty that the designer has about the attack the system is subject to, and, on the other hand, with the uncertainty of the adversary about the target system (i.e., the system it aims at defeating). Game theory has recently been proposed as a way to study the strategic interaction between the choices made by the system designer and the adversary. Among the fields wherein such an approach has been used, we mention steganalysis [2,3], watermarking [4], intrusion detection [5] and adversarial machine learning [6,7]. With regard to binary detection, game theory and information theory have been combined to address the problem of adversarial detection, especially in the field of digital watermarking (see, for instance, [4,8,9,10]). In all these works, the problem of designing watermarking codes that are robust to intentional attacks is studied as a game between the information hider and the attacker.

An attempt to develop a general theory for the binary hypothesis testing problem in the presence of an adversary is made in [11]. Specifically, in [11], the general problem of binary decision under adversarial conditions is addressed and formulated as a game between two players, the *defender* and the *attacker*, which have conflicting goals. Given two discrete memoryless sources, $P_{0}$ and $P_{1}$, the goal of the defender is to decide whether a given test sequence has been generated by $P_{0}$ (null hypothesis, $H_{0}$) or $P_{1}$ (alternative hypothesis, $H_{1}$). By adopting the Neyman–Pearson approach, the set of strategies the defender can choose from is the set of decision regions for $H_{0}$ ensuring that the false positive error probability is lower than a given threshold. On the other hand, the ultimate goal of the attacker in [11] is to cause a false negative decision, so the attacker acts under $H_{1}$ only. In other words, the attacker modifies a sequence generated by $P_{1}$, in attempt to move it into the acceptance region of $H_{0}$. The attacker is subjected to a distortion constraint, which limits his freedom in doing so. Such a struggle between the defender and the attacker is modeled in [11] as a competitive zero-sum game; the asymptotic equilibrium, i.e., the equilibrium when the length of the observed sequence tends to infinity, is derived under the assumption that the defender bases his decision on the analysis of first-order statistics only. In this respect, the analysis conducted in [11] extends the one of [12] to the adversarial scenario. Some variants of this attack-detection game have also been studied; for instance, in [13], the setting is extended to the case where the sources are known to neither the defender nor the attacker, while the training data from both sources are available to both parties. Within this framework, the case where part of the training data available to the defender is corrupted by the attacker has also been studied (see [14]).

The assumption that the attacker is active only under $H_{1}$ stems from the observation that in many cases $H_{0}$ corresponds to a kind of *normal* or *safe* situation and its rejection corresponds to revealing the presence of a threat or that something anomalous is happening. This is the case, for instance, in intrusion detection systems, tampering detection, target detection in radar systems, steganalysis and so on. In these cases, the goal of the attacker is to avoid the possibility that the monitoring system raises an alarm by rejecting the hypothesis $H_{0}$, while having no incentive to act under $H_{0}$. Moreover, in many cases, the attack is not even present under $H_{0}$ or it does not have the possibility of manipulating the observations the detector relies on when $H_{0}$ holds.

In many other cases, however, it is reasonable to assume that the attacker is active under both hypotheses with the goal of causing both false positive and false negative detection errors. As an example, we may consider the case of a radar target detection system, where the defender wishes to distinguish between the presence and the absence of a target, by considering the presence of a hostile jammer. To maximize the damage caused by his actions, the jammer may decide to work under both the hypotheses: when $H_{1}$ holds, to avoid that the defender detects the presence of the target, and in the $H_{0}$ case, to increase the number of false alarms inducing a waste of resources deriving from the adoption of possibly expensive countermeasures even when they are not needed. In a completely different scenario, we may consider an image forensic system aiming at deciding whether a certain image has been shot by a given camera, for instance because the image is associated to a crime such as child pornography or terrorism. Even in this case, the attacker may be interested in causing a missed detection event to avoid that he is accused of the crime associated to the picture, or to induce a false alarm to accuse an innocent victim. Other examples come from digital watermarking, where an attacker can be interested in either removing or injecting the watermark from an image or a video, to redistribute the content without any ownership information or in such a way to support a false copyright statement [15], and network intrusion detection systems [16], wherein the adversary may try to both avoid the detection of the intrusion by manipulating a malicious traffic, and to implement an overstimulation attack [17,18] to cause a denial of service failure.

According to the scenario at hand, the attacker may be aware of the hypothesis under which it is operating (hypothesis-aware attacker) or not (hypothesis-unaware attacker). While the setup considered in this paper can be used to address both cases, we focus mainly on the case of an hypothesis-aware attacker, since this represents a worst-case assumption for the defender. In addition, the case of an hypothesis-unaware attacker can be handled as a special case of the more general case of an aware attacker subject to identical constraints under the two hypotheses.

With the above ideas in mind, in this paper, we consider the game-theoretic formulation of the defender–attacker interaction when the attacker acts under both hypotheses. We refer to this scenario as a detection game with a *fully active attacker*. By contrast, when the attacker acts under hypothesis $H_{1}$ only (as in [11,13]), it is referred to as a *partially active attacker*. As we show, the game in the partially active case turns out to be a special case of the game with fully active, hypothesis-aware, attacker. Accordingly, the hypothesis-aware fully active scenario forms a unified framework that includes the hypothesis-unaware case and the partially active scenario as special cases.

We define and solve two versions of the *detection game with fully active attackers*, corresponding to two different formulations of the problem: a Neyman–Pearson formulation and a Bayesian formulation. Another difference with respect to [11] is that here the players are allowed to adopt randomized strategies. Specifically, the defender can adopt a *randomized decision* strategy, while in [11] the defender’s strategies are confined to deterministic decision rules. As for the attack, it consists of the application of a *channel*, whereas in [11] it is confined to the application of a deterministic function. Moreover, the partially active case of [11] can easily be obtained as a special case of the fully active case considered here. The problem of solving the game and then finding the optimal detector in the adversarial setting is not trivial and may not be possible in general. Thus, we limit the complexity of the problem and make the analysis tractable by confining the decision to depend on a given set of statistics of the observation. Such an assumption, according to which the detector has access to a limited set of empirical statistics of the sequence, is referred to as *limited resources* assumption (see [12] for an introduction on this terminology). In particular, as already done in related literature [11,13,14], we limit the detection resources to first-order statistics, which are known to be a sufficient statistic for memoryless systems (Section 2.9, [19]). In the setup studied in this paper, the sources are indeed assumed to be memoryless, however one might still be concerned regarding the sufficiency of first-order statistics in our setting, since the attack channel is not assumed memoryless in the first place. Forcing, nonetheless, the defender to rely on first-order statistics is motivated mainly by its simplicity. In addition, the use of first-order statistics is common in a number of application scenarios even if the source under analysis is not memoryless. In image forensics, for instance, several techniques have been proposed which rely on the analysis of the image histogram or a subset of statistics derived from it, e.g., for the detection of contrast enhancement [20] or cut-and-paste [21] processing. As another example, the analysis of statistics derived from the histograms of block-DCT coefficients is often adopted for detecting both single and multiple JPEG compression [22]. More generally, we observe that the assumption of limited resources is reasonable in application scenarios where the detector has a small computational power. Having said that, it should be also emphasized that the analysis in this work can be extended to richer sets of empirical statistics, e.g., higher-order statistics (see the Conclusions Section for a more elaborated discussion on this point).

As a last note, we observe that, although we limit ourselves to memoryless sources, our results can be easily extended to more general models (e.g., Markov sources), as long as a suitable extension of the method of types is available.

One of the main results of this paper is the characterization of an attack strategy that is both *dominant* (i.e., optimal no matter what the defense strategy is), and *universal*, i.e., independent of the (unknown) underlying sources. Moreover, this optimal attack strategy turns out to be the same under both hypotheses, thus rendering the distinction between the hypothesis-aware and the hypothesis-unaware scenarios completely inconsequential. In other words, the optimal attack strategy is universal, not only with respect to uncertainty in the source statistics under either hypothesis, but also with respect to the unknown hypothesis in the hypothesis-unaware case. Moreover, the optimal attack is the same for both the Neyman–Pearson and Bayesian games. This result continues to hold also for the partially active case, thus marking a significant difference with respect to previous works [11,13], where the existence of a dominant strategy wasestablished with regard to the defender only.

Some of our results (in particular, the derivation of the equilibrium point for both the Neyman–Pearson and the Bayesian games) have already appeared mostly without proofs in [23]. Here, we provide the full proofs of the main theorems, evaluate the payoff at equilibrium for both the Neyman–Pearson and Bayesian games and include the analysis of the ultimate performance of the games. Specifically, we characterize the so called indistinguishability region (to be defined formally in Section 6), namely the set of the sources for which it is not possible to attain strictly positive exponents for both false positive and false negative probabilities under the Neyman–Pearson and the Bayesian settings. Furthermore, the setup and analysis presented in [23] is extended by considering a more general case in which the maximum allowed distortion levels the attacker may introduce under the two hypotheses are different.

The paper is organized as follows. In Section 2, we establish the notation and introduce the main concepts. In Section 3, we formalize the problem and define the detection game with a fully active adversary for both the Neyman–Pearson and the Bayesian games, and then prove the existence of a dominant and universal attack strategy. The complete analysis of the Neyman–Pearson and Bayesian detection games, namely, the study of the equilibrium point of the game and the computation of the payoff at the equilibrium, are carried out in Section 4 and Section 5, respectively. Finally, Section 6 is devoted to the analysis of the best achievable performance of the defender and the characterization of the source distinguishability.

## 2. Notation and Definitions

Throughout the paper, random variables are denoted by capital letters and specific realizations are denoted by the corresponding lower case letters. All random variables that denote signals in the system are assumed to have the same finite alphabet, denoted by A. Given a random variable *X* and a positive integer *n*, we denote by $X=(X_{1},X_{2},\ldots ,X_{n})$, $X_{i}\in A$, i=1,2,…,n, a sequence of *n* independent copies of ***x***. According to the above-mentioned notation rules, a specific realization of ***X*** is denoted by $x=(x_{1},x_{2},\ldots ,x_{n})$. Sources are denoted by the letter *P*. Whenever necessary, we subscript *P* with the name of the relevant random variables: given a random variable ***x***, $P_{X}$ denotes its probability mass function (PMF). Similarly, $P_{XY}$ denotes the joint PMF of a pair of random variables, (X,Y). For two positive sequences, $\left\{a_{n}\right\}$ and $\left\{b_{n}\right\}$, the notation $a_{n}\overset{\cdot }{=}b_{n}$ stands for exponential equivalence, i.e., $\lim_{n\to \infty }1/n\ln\left(a_{n}/b_{n}\right)=0$, and $a_{n}\overset{\cdot }{\leq }b_{n}$ designates that $\underset{n\to \infty }{lim sup}1/n\ln\left(a_{n}/b_{n}\right)\leq 0$.

For a given real *s*, we denote ${\left[s\right]}_{+}\overset{▵}{=}\max\left\{s,0\right\}$. We use notation U(·) for the Heaviside step function.

The type of a sequence $x\in A^{n}$ is defined as the empirical probability distribution ${\hat{P}}_{x}$, that is, the vector $\left\{{\hat{P}}_{x}\left(x\right),\;x\in A\right\}$ of the relative frequencies of the various alphabet symbols in ***x***. A type class T(x) is defined as the set of all sequences having the same type as ***x***. When we wish to emphasize the dependence of T(x) on ${\hat{P}}_{x}$, we use the notation $T({\hat{P}}_{x})$. Similarly, given a pair of sequences (x,y), both of length *n*, the joint type class T(x,y) is the set of sequence pairs $\left\{\left(x^{\prime },y^{\prime }\right)\right\}$ of length *n* having the same empirical joint probability distribution (or joint type) as (x,y), ${\hat{P}}_{xy}$, and the conditional type class T(y|x) is the set of sequences $\left\{y^{\prime }\right\}$ with ${\hat{P}}_{xy^{\prime }}={\hat{P}}_{xy}$.

Regarding information measures, the entropy associated with ${\hat{P}}_{x}$, which is the empirical entropy of ***x***, is denoted by ${\hat{H}}_{x}\left(X\right)$. Similarly, ${\hat{H}}_{xy}\left(X,Y\right)$ designates the empirical joint entropy of ***x*** and ***y***, and ${\hat{H}}_{xy}\left(X|Y\right)$ is the conditional joint entropy. We denote by D(P∥Q) the Kullback–Leibler (K-L) divergence between two sources, *P* and *Q*, with the same alphabet (see [19]).

Finally, we use the letter *A* to denote an attack channel; accordingly, A(y|x) is the conditional probability of the channel output ***y*** given the channel input ***x***. Given a permutation-invariant distortion function $d:A^{n}\times A^{n}\to {I\;R}^{+}$ (a permutation-invariant distortion function d(x,y) is a distortion function that is invariant if the same permutation is applied to both ***x*** and ***y***) and a maximum distortion Δ, we define the class $C_{\Delta }$ of admissible channels $\left\{A\left(y|x\right),\;x,y\in A^{n}\right\}$ as those that assign zero probability to every ***y*** with d(x,y)>nΔ.

### 2.1. Basics of Game Theory

For the sake of completeness, we introduce some basic definitions and concepts of game theory. A two-player game is defined as a quadruple $\left(S_{1},S_{2},u_{1},u_{2}\right)$, where $S_{1}=\left\{s_{1,1}\cdots s_{1,n_{1}}\right\}$ and $S_{2}=\left\{s_{2,1}\cdots s_{2,n_{2}}\right\}$ are the sets of strategies from which the first and second player can choose, respectively, and $u_{l}\left(s_{1,i},s_{2,j}\right),l=1,2$, is the payoff of the game for player *l*, when the first player chooses the strategy $s_{1,i}$ and the second one chooses $s_{2,j}$. Each player aims at maximizing its payoff function. A pair of strategies $\left(s_{1,i},s_{2,j}\right)$ is called a *profile*. When $u_{1}\left(s_{1,i},s_{2,j}\right)+u_{2}\left(s_{1,i},s_{2,j}\right)=0$, the game is said to be a *zero-sum game*. For such games, the payoff of the game $u(s_{1,i},s_{2,j})$ is usually defined by adopting the perspective of one of the two players: that is, $u\left(s_{1,i},s_{2,j}\right)=u_{1}\left(s_{1,i},s_{2,j}\right)=-u_{2}\left(s_{1,i},s_{2,j}\right)$ if the defender’s perspective is adopted or vice versa. The sets $S_{1}$ and $S_{2}$ and the payoff functions are assumed known to both players. In addition, we consider *strategic games*, i.e., games in which the players choose their strategies ahead of time, without knowing the strategy chosen by the opponent.

A common goal in game theory is to determine the existence of *equilibrium points*, i.e., profiles that in *some sense* represent a *satisfactory* choice for both players [24]. The most famous notion of equilibrium is due to Nash [25]. A profile is said to be a *Nash equilibrium* if no player can improve its payoff by changing its strategy unilaterally.

Despite its popularity, the practical meaning of Nash equilibrium is often unclear, since there is no guarantee that the players will end up playing at the Nash equilibrium. A particular kind of games for which stronger forms of equilibrium exist are the so-called *dominance solvable* games [24]. The concept of dominance-solvability is directly related to the notion of strict dominance and dominated strategies. In particular, a strategy is said to be *strictly dominant* for one player if it is the best strategy for this player, i.e., the strategy that maximizes the payoff, no matter what the strategy of the opponent may be. Similarly, we say that a strategy $s_{l,i}$ is *strictly dominated* by strategy $s_{l,j}$, if the payoff achieved by player *l* choosing $s_{l,i}$ is always lower than that obtained by playing $s_{l,j}$, regardless of the strategy of the other player. Recursive elimination of dominated strategies is a common technique for solving games. In the first step, all the dominated strategies are removed from the set of available strategies, since no *rational* player (in game theory, a rational player is supposed to act in a way that maximizes its payoff) would ever use them. In this way, a new, smaller game is obtained. At this point, some strategies that were not dominated before, may become dominated in the new, smaller version of the game, and hence are eliminated as well. The process goes on until no dominated strategy exists for either player. A *rationalizable equilibrium* is any profile which survives the iterated elimination of dominated strategies [26,27]. If at the end of the process only one profile is left, the remaining profile is said to be the *only rationalizable equilibrium* of the game, which is also the only Nash equilibrium point. Dominance solvable games are easy to analyze since, under the assumption of rational players, we can anticipate that the players will choose the strategies corresponding to the unique rationalizable equilibrium. Another, related, interesting notion of equilibrium is that of *dominant equilibrium*. A dominant equilibrium is a profile that corresponds to dominant strategies for both players and is the strongest kind of equilibrium that a strategic game may have.

## 3. Detection Game with Fully Active Attacker

### 3.1. Problem Formulation

Given two discrete memoryless sources, $P_{0}$ and $P_{1}$, defined over a common finite alphabet A, we denote by $x=\left(x_{1},\ldots ,x_{n}\right)\in A^{n}$ a sequence emitted by one of these sources. The sequence ***x*** is available to the attacker. Let $y=\left(y_{1},y_{2},\ldots ,y_{n}\right)\in A^{n}$ denote the sequence observed by the defender: when an attack occurs under both $H_{0}$ and $H_{1}$, the observed sequence ***y*** is obtained as the output of an attack channel fed by ***x***.

In principle, we must distinguish between two cases: in the first, the attacker is aware of the underlying hypothesis (hypothesis-aware attacker), whereas, in the second case, it is not (hypothesis-unaware attacker). In the hypothesis-aware case, the attack strategy is defined by two different conditional probability distributions, i.e., two different attack channels: $A_{0}\left(y|x\right)$, applied when $H_{0}$ holds, and $A_{1}\left(y|x\right)$, applied under $H_{1}$. Let us denote by $Q_{i}\left(\cdot \right)$ the PMF of ***y*** under $H_{i}$, i=0,1. The attack induces the following PMFs on ***y***: $Q_{0}\left(y\right)=\sum_{x}P_{0}\left(x\right)A_{0}\left(y|x\right)$ and $Q_{1}\left(y\right)=\sum_{x}P_{1}\left(x\right)A_{1}\left(y|x\right)$.

Clearly, in the hypothesis-unaware case, the attacker will apply the same channel under $H_{0}$ and $H_{1}$, that is, $A_{0}=A_{1}$, and we denote the common attack channel simply by *A*. Throughout the paper, we focus on the hypothesis-aware case as, in view of this formalism, the hypothesis-unaware case is just a special case. Obviously, the partially active case, where no attack occurs under $H_{0}$ can be seen as a degenerate case of the hypothesis-aware fully active one, where $A_{0}$ is the identity channel *I*.

Regarding the defender, we assume a randomized decision strategy, defined by $\Phi (H_{i}|y)$, which designates the probability of deciding in favor of $H_{i}$, i=0,1, given ***y***. Accordingly, the probability of a *false positive* (FP) decision error is given by

(1) $$P_{\mathrm{FP}}\left(\Phi ,A_{0}\right)=\sum_{y}Q_{0}\left(y\right)\Phi \left(H_{1}|y\right),$$

and similarly, the *false negative* (FN) probability assumes the form:

(2) $$P_{\mathrm{FN}}\left(\Phi ,A_{1}\right)=\sum_{y}Q_{1}\left(y\right)\Phi \left(H_{0}|y\right).$$

As in [11], due to the limited resources assumption, the defender makes a decision based on first-order empirical statistics of ***y***, which implies that Φ(·|y) depends on ***y*** only via its type class T(y).

Concerning the attack, to limit the amount of distortion, we assume a distortion constraint. In the hypothesis-aware case, we allow the attacker different distortion levels, $\Delta_{0}$ and $\Delta_{1}$, under $H_{0}$ and $H_{1}$, respectively. Then, $A_{0}\in C_{\Delta_{0}}$ and $A_{1}\in C_{\Delta_{1}}$, where, for simplicity, we assume that a common (permutation-invariant) distortion function d(·,·) is adopted in both cases.

**Figure 1:**
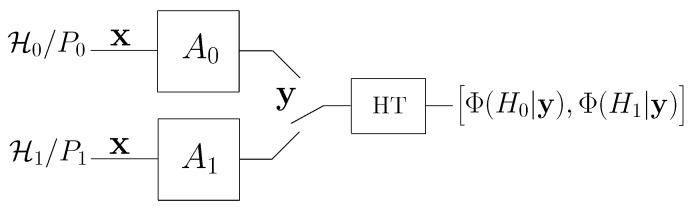
Schematic representation of the adversarial setup considered in this paper. In the case of partially active attacker, channel $A_{0}$ corresponds to the identity channel.

Figure 1 provides a block diagram of the system with a fully active attacker studied in this paper.

### 3.2. Definition of the Neyman–Pearson and Bayesian Games

One of the difficulties associated with the fully active setting is that, in the presence of a fully active attacker, both the FP and FN probabilities depend on the attack channels. We therefore consider two different approaches, which lead to different formulations of the detection game: in the first, the detection game is based on the Neyman–Pearson criterion, and, in the second one, the Bayesian approach is adopted.

For the Neyman–Pearson setting, we define the game by assuming that the defender adopts a conservative approach and imposes an FP constraint pertaining to the worst-case attack under $H_{0}$.

**Definition** **1.** *The Neyman–Pearson detection game is a zero-sum, strategic game defined as follows.*

- *The set $S_{D}$ of strategies allowed to the defender is the class of randomized decision rules {Φ} that satisfy*
  **(i)**  $\Phi (H_{0}|y)$ depends on **y** only via its type.
  **(ii)**  $\max_{A_{0}\in C_{\Delta_{0}}}P_{FP}\left(\Phi ,A_{0}\right)\leq e^{-n\lambda }$ for a prescribed constant λ>0, independent of n.
- The set $S_{A}$ of strategies allowed to the attacker is the class of pairs of attack channels $\left(A_{0},A_{1}\right)$ such that $A_{0}\in C_{\Delta_{0}}$, $A_{1}\in C_{\Delta_{1}}$; that is, $S_{A}=C_{\Delta_{0}}\times C_{\Delta_{1}}$.
- The payoff of the game is $u\left(\Phi ,A_{1}\right)=P_{\mathrm{FN}}\left(\Phi ,A_{1}\right)$; the attacker is in the quest of maximizing $u(\Phi ,A_{1})$ whereas the defender wishes to minimize it.

In the above definition, we require that the FP probability decays exponentially fast with *n*, with an exponential rate *at least* as large as λ. Such a requirement is relatively strong, its main consequence being that the strategy used by the attacker under $H_{0}$ is irrelevant, in that the payoff is the same whichever is the channel $A_{0}\in C_{\Delta_{0}}$ played by the attacker. It is worth observing that, to be more general, we could have defined the problem as a non-zero sum game, where the defender has payoff $u_{D}\left(\phi ,A_{1}\right)=-P_{\mathrm{FN}}\left(\Phi ,A_{1}\right)$, whereas for the attacker we consider a payoff function of the form $u_{A}\left(\phi ,\left(A_{0},A_{1}\right)\right)=\beta P_{\mathrm{FP}}\left(\Phi ,A_{0}\right)+\gamma P_{\mathrm{FN}}\left(\Phi ,A_{1}\right)$, for some positive constant β and γ. As is made clear in the following section, this non-zero sum version of the game has the same equilibrium strategies of the zero-sum game defined above.

In the case of partially active attack (see the formulation in [23]), the FP probability does not depend on the attack but on the defender only; accordingly, the constraint imposed by the defender in the above formulation becomes $P_{\mathrm{FP}}\left(\Phi \right)\leq e^{-n\lambda }$. Regarding the attacker, we have $S_{A}\equiv C_{0}\times C_{\Delta_{1}}$, where $C_{0}$ is a singleton that contains the identity channel only.

Another version of the detection game is defined by assuming that the defender follows a less conservative approach, that is, the Bayesian approach, and he tries to minimize a particular Bayes risk.

**Definition** **2.** *The Bayesian detection game is a zero-sum, strategic game defined as follow.*

- The set $S_{D}$ of strategies allowed to the defender is the class of the randomized decision rules {Φ} where $\Phi (H_{0}|y)$ depends on **y** only via its type.
- The set $S_{A}$ of strategies allowed to the attacker is $S_{A}=C_{\Delta_{0}}\times C_{\Delta_{1}}$.
- *The payoff of the game is*
  (3) $$u\left(\Phi ,\left(A_{0},A_{1}\right)\right)=P_{\mathrm{FN}}\left(\Phi ,A_{1}\right)+e^{an}P_{FP}\left(\Phi ,A_{0}\right),$$
  *for some constant a, independent of n.*

We observe that, in the definition of the payoff, the parameter *a* controls the tradeoff between the two terms in the exponential scale; whenever possible, the optimal defense strategy is expected to yield error exponents that differ exactly by *a*, so as to balance the contributions of the two terms of Equation (3).

Notice also that, by defining the payoff as in Equation (3), we are implicitly considering for the defender only the strategies Φ(·|y) such that $P_{\mathrm{FP}}\left(\Phi ,A_{0}\right)\overset{\cdot }{\leq }e^{-an}$. In fact, any strategy that does not satisfy this inequality yields a payoff u>1 that cannot be optimal, as it can be improved by always deciding in favor of $H_{0}$ regardless of ***y*** (u=1).

As in [11], we focus on the asymptotic behavior of the game as *n* tends to infinity. In particular, we are interested in the exponents of the FP and FN probabilities, defined as follows:

(4) $$\varepsilon_{\mathrm{FP}}=-\underset{n\to \infty }{lim sup}\frac{1}{n}\ln P_{\mathrm{FP}}\left(\Phi ,A_{0}\right);\;\varepsilon_{\mathrm{FN}}=-\underset{n\to \infty }{lim sup}\frac{1}{n}\ln P_{\mathrm{FN}}\left(\Phi ,A_{1}\right).$$

We say that a strategy is *asymptotically optimal* (or *dominant*) if it is optimal (dominant) with respect to the asymptotic exponential decay rate (or the exponent, for short) of the payoff.

### 3.3. Asymptotically Dominant and Universal Attack

In this subsection, we characterize an attack channel that, for both games, is asymptotically dominant and universal, in the sense of being independent of the unknown underlying sources. This result paves the way to the solution of the two games.

Let *u* denote a generic payoff function of the form

(5) $$u=\gamma P_{\mathrm{FN}}\left(\Phi ,A_{1}\right)+\beta P_{\mathrm{FP}}\left(\Phi ,A_{0}\right),$$

where β and γ are given positive constants, possibly dependent on *n*.

We notice that the payoff of the Neyman–Pearson and Bayesian games defined in the previous section can be obtained as particular cases: specifically, γ=1 and β=0 for the Neyman–Pearson game and γ=1 and $\beta =e^{an}$ for the Bayesian one.

**Theorem** **1.** Let $c_{n}\left(x\right)$ denote the reciprocal of the total number of conditional type classes {T(y|x)} that satisfy the constraint d(x,y)≤nΔ for a given Δ>0, namely, admissible conditional type classes (from the method of the types, it is known that $1\geq c_{n}\left(x\right)\geq {\left(n+1\right)}^{-|A|\cdot (|A|-1)}$ for any ***x*** [19]).

*Define:*

(6) $$A_{\Delta }^{*}\left(y|x\right)=\left\{\begin{array}{cc} \frac{c_{n}\left(x\right)}{\left|T(y|x)\right|} & d(x,y)\leq n\Delta \\ 0 & elsewhere \end{array}\right..$$

Among all pairs of channels $\left(A_{0},A_{1}\right)\in S_{A}$, the pair $\left(A_{\Delta_{0}}^{*},A_{\Delta_{1}}^{*}\right)$ minimizes the asymptotic exponent of u for every $P_{0}$ and $P_{1}$, every γ,β≥0 and every permutation-invariant $\Phi (H_{0}|\cdot )$.

**Proof.**  We first focus on the attack under $H_{1}$ and therefore on the FN probability.

Consider an arbitrary channel $A_{1}\in C_{\Delta_{1}}$. Let $\Pi :A^{n}\to A^{n}$ denote a permutation operator that permutes any member of $A^{n}$ according to a given permutation matrix and let

(7) $$A_{\Pi }\left(y|x\right)\overset{▵}{=}A_{1}\left(\Pi y|\Pi x\right).$$

Since the distortion function is assumed to be permutation-invariant, the channel $A_{\Pi }\left(y|x\right)$ introduces the same distortion as $A_{1}$, and hence it satisfies the distortion constraint. Due to the memorylessness of $P_{1}$ and the assumption that $\Phi (H_{0}|\cdot )$ belongs to $S_{D}$ (i.e., that $\Phi (H_{0}|\cdot )$ depends on the observed sequence via its type), both $P_{1}\left(y\right)$ and $\Phi (H_{0}|y)$ are invariant to permutations on ***y***. Then, we have:

(8) $$\begin{array}{ccc} P_{\mathrm{FN}}\left(\Phi ,A_{\Pi }\right) & = & \sum_{x,y}P_{1}\left(x\right)A_{\Pi }\left(y|x\right)\Phi \left(H_{0}|y\right)\\ & = & \sum_{x,y}P_{1}\left(x\right)A_{1}\left(\Pi y|\Pi x\right)\Phi \left(H_{0}|y\right)\\ & = & \sum_{x,y}P_{1}\left(\Pi x\right)A_{1}\left(\Pi y|\Pi x\right)\Phi \left(H_{0}|\Pi y\right)\\ & = & \sum_{x,y}P_{1}\left(x\right)A_{1}\left(y|x\right)\Phi \left(H_{0}|y\right)\\ & = & P_{\mathrm{FN}}\left(\Phi ,A_{1}\right), \end{array}$$

thus $P_{\mathrm{FN}}\left(\Phi ,A_{1}\right)=P_{\mathrm{FN}}\left(\Phi ,\bar{A}\right)$, where we have defined

(9) $$\bar{A}\left(y|x\right)=\frac{1}{n!}\sum_{\Pi }A_{\Pi }\left(y|x\right)=\frac{1}{n!}\sum_{\Pi }A_{1}\left(\Pi y|\Pi x\right),$$

which also introduces the same distortion as $A_{1}$. Now, notice that this channel assigns the same conditional probability to all sequences in the same conditional type class T(y|x). To see why this is true, we observe that any sequence $y^{\prime }\in T\left(y|x\right)$ can be seen as being obtained from ***y*** through the application of a permutation $\Pi^{\prime }$, which leaves ***x*** unaltered. Then, we have:

(10) $$\begin{array}{cc} \bar{A}\left(y^{\prime }|x\right) & =\bar{A}\left(\Pi^{\prime }y|\Pi^{\prime }x\right)=\frac{1}{n!}\sum_{\Pi }A_{1}\left(\Pi \left(\Pi^{\prime }y\right)|\Pi \left(\Pi^{\prime }x\right)\right)\\ & =\frac{1}{n!}\sum_{\Pi }A_{1}\left(\Pi y|\Pi x\right)=\bar{A}\left(y|x\right). \end{array}$$

Therefore, since the probability assigned by $\bar{A}$ to the sequences in T(y|x) is surely less than or equal to 1, we argue that

(11) $$\begin{array}{ccc} \bar{A}\left(y|x\right) & \overset{\cdot }{\leq } & \left\{\begin{array}{cc} \frac{1}{\left|T(y|x)\right|} & d(x,y)\leq n\Delta \\ 0 & \mathrm{elsewhere} \end{array}\right.\\ & = & \frac{A_{\Delta_{1}}^{*}\left(y|x\right)}{c_{n}\left(x\right)}\\ & \leq & {\left(n+1\right)}^{\left|A|\cdot (|A|-1\right)}A_{\Delta_{1}}^{*}\left(y|x\right), \end{array}$$

which implies that, $P_{\mathrm{FN}}\left(\Phi ,\bar{A}\right)\overset{\cdot }{\leq }{\left(n+1\right)}^{\left|A|\cdot (|A|-1\right)}P_{\mathrm{FN}}\left(A_{\Delta_{1}}^{*},\Phi \right)$.

Then,

(12) $$P_{\mathrm{FN}}\left(\Phi ,A_{1}\right)\overset{\cdot }{\leq }{\left(n+1\right)}^{\left|A|\cdot (|A|-1\right)}P_{\mathrm{FN}}\left(A_{\Delta_{1}}^{*},\Phi \right),$$

or equivalently

(13) $$P_{\mathrm{FN}}\left(\Phi ,A_{\Delta_{1}}^{*}\right)\overset{\cdot }{\geq }{\left(n+1\right)}^{-|A|\cdot (|A|-1)}P_{\mathrm{FN}}\left(A_{1},\Phi \right).$$

We conclude that $A_{\Delta_{1}}^{*}$ minimizes the error exponent of $P_{\mathrm{FN}}\left(\Phi ,A_{1}\right)$ across all channels in $C_{\Delta_{1}}$ and for every $\Phi \in S_{D}$, regardless of $P_{1}$.

A similar argument applies to the FP probability for the derivation of the optimal channel under $H_{0}$; that is, from the memorylessness of $P_{0}$ and the permutation-invariance of $\Phi (H_{1}|\cdot )$, we have:

(14) $$P_{\mathrm{FP}}\left(\Phi ,A_{\Delta_{0}}^{*}\right)\geq {\left(n+1\right)}^{-|A|\cdot (|A|-1)}P_{\mathrm{FP}}\left(A_{0},\Phi \right),$$

for every $A_{0}\in C_{\Delta_{0}}$. Accordingly, $A_{\Delta_{0}}^{*}$ minimizes the error exponent of $P_{\mathrm{FP}}\left(\Phi ,A_{0}\right)$. We then have:

(15) $$\begin{array}{c} \gamma P_{\mathrm{FN}}\left(\Phi ,A_{1}\right)+\beta P_{\mathrm{FP}}\left(\Phi ,A_{0}\right)\\ \;\leq {\left(n+1\right)}^{\left|A|\cdot (|A|-1\right)}\left(\gamma P_{\mathrm{FN}}\left(\Phi ,A_{\Delta_{1}}^{*}\right)+\beta P_{\mathrm{FP}}\left(\Phi ,A_{\Delta_{0}}^{*}\right)\right)\\ \;≐\gamma P_{\mathrm{FN}}\left(\Phi ,A_{\Delta_{1}}^{*}\right)+\beta P_{\mathrm{FP}}\left(\Phi ,A_{\Delta_{0}}^{*}\right), \end{array}$$

for every $A_{0}\in C_{\Delta_{0}}$ and $A_{1}\in C_{\Delta_{1}}$. Notice that, since the asymptotic equality is defined in the exponential scale, Equation (15) holds no matter what the values of β and γ are, including values that depend on *n*. Hence, the pair of channels $\left(A_{\Delta_{0}}^{*},A_{\Delta_{1}}^{*}\right)$ minimizes the asymptotic exponent of *u* for any permutation-invariant decision rule $\Phi (H_{0}|\cdot )$ and for any γ,β≥0. ☐

**Figure 2:**
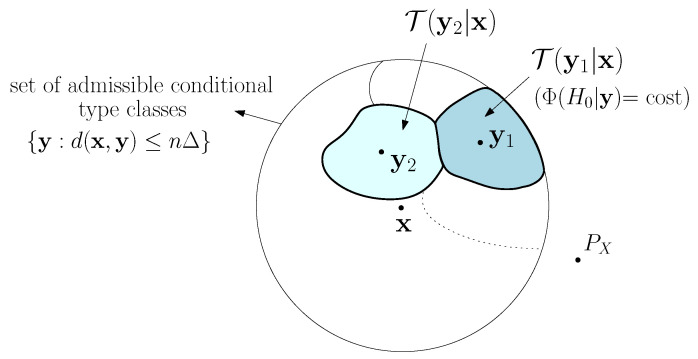
Graphical interpretation of the behavior of the attack channel $A_{\Delta }^{*}$. The number of admissible conditional type classes is polynomial in *n*, that is $\left\{y:d\left(x,y\right)\leq n\Delta \right\}=\underset{i\in p(n)}{⋃}T\left(y_{i}|x\right)$ where p(n) is a polynomial of *n*.

According to Theorem 1, for every zero-sum game with payoff function of the form in Equation (5), if Φ is permutation-invariant, the pair of attack channels which is the most favorable to the attacker is $\left(A_{\Delta_{0}}^{*},A_{\Delta_{1}}^{*}\right)$, which does not depend on Φ. Then, the optimal attack strategy $\left(A_{\Delta_{0}}^{*},A_{\Delta_{1}}^{*}\right)$ is *dominant*. The intuition behind the attack channel in Equation (6) is illustrated in Figure 2 and explained below. Given ***x***, generated by a source $P_{X}$, the set {y:d(x,y)≤nΔ} corresponds to a set of conditional type classes (for a permutation-invariant distortion function, d(x,y) is the same for every y∈T(y|x)). We say that a conditional type class is *admissible* if it belongs to this set. Then, to generate ***y*** which causes a detection error with the prescribed maximum allowed distortion, the attacker cannot do any better than randomly selecting an admissible conditional type class according to the uniform distribution and then choosing at random ***y*** within this conditional type class. Since the number of conditional type classes is only polynomial in *n*, the random choice of the conditional type class does not affect the exponent of the error probabilities; besides, since the decision is the same for all sequences within the same conditional type class, the choice of ***y*** within that conditional type class is immaterial.

As an additional result, Theorem 1 states that, whenever an adversary aims at maximizing a payoff function of the form Equation (5), and as long as the defense strategy is confined to the analysis of the first-order statistics, the (asymptotically) optimal attack strategy is *universal* with respect to the sources $P_{0}$ and $P_{1}$, i.e., it depends neither on $P_{0}$ nor on $P_{1}$.

We observe that, if $\Delta_{0}=\Delta_{1}=\Delta$, the optimal attack consists of applying the same channel $A_{\Delta }^{*}$ regardless of the underlying hypothesis and then the optimal attack strategy is *fully-universal*: the attacker needs to know neither the sources ($P_{0}$ and $P_{1}$), nor the underlying hypothesis. In this case, it becomes immaterial whether the attacker is aware or unaware of the true hypothesis. As a consequence of this property, in the hypothesis-unaware case, when the attacker applies the same channel under both hypotheses, subject to a fixed maximum distortion Δ, the optimal channel remains $A_{\Delta }^{*}$.

According to Theorem 1, for the partially active case, there exists an (asymptotically) dominant and universal attack channel. This result marks a considerable difference with respect to the results of [11], where the optimal deterministic attack function is found by using the rationalizability argument, that is, by exploiting the existence of a dominant defense strategy, and it is hence neither dominant nor universal.

Finally, we point out that, similar to [11], although the sources are memoryless and only first-order statistics are used by the defender, the output probability distributions induced by the attack channel $A_{\Delta }^{*}$, namely $Q_{0}$ and $Q_{1}$, are not necessarily memoryless.

## 4. The Neyman–Pearson Detection Game

In this section, we study the detection game with a fully active attacker in the Neyman–Pearson setup as defined in Definition 1. From the analysis of Section 3.3, we already know that there exists a dominant attack strategy. Regarding the defender, we determine the asymptotically optimal strategy regardless of the dominant pair of attack channels; in particular, as shown in Lemma 1, an asymptotically dominant defense strategy can be derived from a detailed analysis of the FP constraint. Consequently, the Neyman–Pearson detection game has a dominant equilibrium.

### 4.1. Optimal Detection and Game Equilibrium

The following lemma characterizes the optimal detection strategy in the Neyman–Pearson setting.

**Lemma** **1.** *For the Neyman–Pearson game of Definition 1, the defense strategy*

(16) $$\begin{array}{cc} \Phi^{*}\left(H_{1}|y\right)\overset{▵}{=} & \exp\left\{-n{\left[\lambda -\min_{x:d\left(x,y\right)\leq n\Delta_{0}}D\left({\hat{P}}_{x}∥P_{0}\right)\right]}_{+}\right\}, \end{array}$$

*is asymptotically dominant for the defender.*

In the special case $\Delta_{0}=0$, $\Phi^{*}\left(H_{1}|y\right)$ in Equation (16) is (asymptotically) equivalent to the Hoeffding test for non-adversarial hypothesis testing [28].

The proof of Lemma 1 appears in Appendix A.1.

We point out that, when the attacker is partially active, it is known from [23] that the optimal defense strategy is

(17) $$\begin{array}{cc} \Phi^{*}\left(H_{1}|y\right)\overset{▵}{=} & \exp\left\{-n{\left[\lambda -D({\hat{P}}_{y}∥P_{0})\right]}_{+}\right\}, \end{array}$$

which is in line with the one in [11] (Lemma 1), where the class of defense strategies is confined to deterministic decision rules.

Intuitively, the extension from Equations (16) and (17) is explained as follows. In the case of fully active attacker, the defender is subject to a constraint on the maximum FP probability over $S_{A}$, that is, the set of the admissible channels $A\in C_{\Delta_{0}}$ (see Definition 1). From the analysis of Section 3.3, channel $A_{\Delta_{0}}^{*}$ minimizes the FP exponent over this set. To satisfy the constraint for a given sequence ***y***, the defender must handle the worst-case value (i.e., the minimum) of $D({\hat{P}}_{x}∥P_{0})$ over all the type classes T(x|y) which satisfy the distortion constraint, or equivalently, all the sequences ***x*** such that $d\left(x,y\right)\leq n\Delta_{0}$.

According to Lemma 1, the best defense strategy is asymptotically dominant. In addition, since $\Phi^{*}$ depends on $P_{0}$ only, and not on $P_{1}$, it is referred to as *semi-universal*.

Concerning the attacker, since the payoff is a special case of Equation (5) with γ=1 and β=0, the optimal pair of attack channels is given by Theorem 1 and corresponds to $\left(A_{\Delta_{0}}^{*},A_{\Delta_{1}}^{*}\right)$.

The following comment is in order. Since the payoff of the game is defined in terms of the FN probability only, it is independent of $A_{0}\in C_{\Delta_{0}}$. Furthermore, since the defender adopts a conservative approach to guarantee the FP constraint for every $A_{0}$, the constraint is satisfied for every $A_{0}$ and therefore all channel pairs of the form $\left(A_{0},A_{\Delta_{1}}^{*}\right)$, $A_{0}\in S_{A}$, are equivalent in terms of the payoff. Accordingly, in the hypothesis-aware case, the attacker can employ any admissible channel under $H_{0}$. In the Neyman–Pearson setting, the sole fact that the attacker is active under $H_{0}$ forces the defender to take countermeasures that make the choice of $A_{0}$ immaterial.

Due to the existence of dominant strategies for both players, we can immediately state the following theorem.

**Theorem** **2.** Consider the Neyman–Pearson detection game of Definition 1. Let $\Phi^{*}$ and $\left(A_{\Delta_{0}}^{*},A_{\Delta_{1}}^{*}\right)$ be the strategies defined in Lemma 1 and Theorem 1, respectively. The profile $\left(\Phi^{*},\left(A_{\Delta_{0}}^{*},A_{\Delta_{1}}^{*}\right)\right)$ is an asymptotically dominant equilibrium of the game.

### 4.2. Payoff at the Equilibrium

In this section, we derive the payoff of the Neyman–Pearson game at the equilibrium of Theorem 2. To do this, we assume an additive distortion function, i.e., $d\left(x,y\right)=\sum_{i=1}^{n}d\left(x_{i},y_{i}\right)$. In this case, d(x,y) can be expressed as $\sum_{ij}n_{xy}\left(i,j\right)d\left(i,j\right)$, where $n_{xy}\left(i,j\right)=n{\hat{P}}_{xy}\left(i,j\right)$ denotes the number of occurrences of the pair $\left(i,j\right)\in A^{2}$ in (x,y). Therefore, the distortion constraint regarding $A_{0}$ can be rewritten as $\sum_{\left(i,j\right)\in A^{2}}{\hat{P}}_{xy}\left(i,j\right)d\left(i,j\right)\leq \Delta_{0}$. A similar formulation holds for $A_{1}$.

Let us define

(18) $${{\tilde{D}}_{\Delta }}^{n}\left({\hat{P}}_{y},P\right)\overset{▵}{=}\min_{\left\{{\hat{P}}_{x|y}:E_{xy}d\left(X,Y\right)\leq \Delta \right\}}D\left({\hat{P}}_{x}∥P\right),$$

where $E_{xy}$ denotes the *empirical expectation*, defined as

(19) $$E_{xy}d\left(X,Y\right)=\sum_{\left(i,j\right)\in A^{2}}{\hat{P}}_{xy}\left(i,j\right)d\left(i,j\right)$$

and the minimization is carried out for a given ${\hat{P}}_{y}$. Accordingly, the strategy in Equation (16) can be rewritten as

(20) $$\Phi^{*}\left(H_{1}|y\right)\overset{▵}{=}\exp\left\{-n{\left[\lambda -{\tilde{D}}_{\Delta_{0}}^{n}\left({\hat{P}}_{y}∥P_{0}\right)\right]}_{+}\right\}.$$

When n→∞, ${\tilde{D}}_{\Delta }^{n}$ becomes (due to the density of rational numbers on the real line, the admissibility set in Equation (18) is dense in that of Equation (21); since the divergence functional is continuous, the sequence ${\left\{{\tilde{D}}_{\Delta }^{n}\left({\hat{P}}_{y},P\right)\right\}}_{n\geq 1}$ tends to ${\tilde{D}}_{\Delta }\left(P_{Y},P\right)$ as n→∞)

(21) $${\tilde{D}}_{\Delta }\left(P_{Y},P\right)\overset{▵}{=}\min_{\left\{P_{X|Y}:E_{XY}d\left(X,Y\right)\leq \Delta \right\}}D\left(P_{X}∥P\right),$$

where $E_{XY}$ denotes expectation with respect to $P_{XY}$.

The definition in Equation (21) can be stated for any PMF $P_{Y}$ in the probability simplex in $R^{\left|A\right|}$. Note that the minimization problem in Equation (21) has a unique solution as it is a convex program.

The function ${\tilde{D}}_{\Delta }$ has an important role in the remaining part of the paper, especially in the characterization of the asymptotic behavior of the games. To draw a parallelism, ${\tilde{D}}_{\Delta }$ plays a role similar to that of the Kullback–Leibler divergence D in classical detection theory for the non-adversarial case.

The basic properties of the functional ${\tilde{D}}_{\Delta }\left(P_{Y},P\right)$ are the following: (i) it is continuous in $P_{Y}$; and (ii) it has convex level sets, i.e., the set $\left\{P_{Y}:{\tilde{D}}_{\Delta }\left(P_{Y},P\right)\leq t\right\}$ is convex for every t≥0. Point (ii) is a consequence of the following property, which is useful for proving some of the results in the sequel (in particular, Theorems 3, 7 and 8).

**Property** **1.** The function ${\tilde{D}}_{\Delta }\left(P_{Y},P\right)$ is convex in $P_{Y}$ for every fixed P.

The proof follows from the convexity of the divergence functional (see Appendix A.2).

Using the above definitions, the equilibrium payoff is given by the following theorem:

**Theorem** **3.** Let the Neyman–Pearson detection game be as in Definition 1. Let $\left(\Phi^{*},\left(A_{\Delta_{0}}^{*},A_{\Delta_{1}}^{*}\right)\right)$ be the equilibrium profile of Theorem 2. Then,

(22) $$\begin{array}{ccc} \varepsilon_{\mathrm{FN}}\left(\lambda \right) & = & -\lim_{n\to \infty }\frac{1}{n}\ln P_{\mathrm{FN}}\left(\Phi^{*},A_{\Delta_{1}}^{*}\right)\\ & = & \min_{P_{Y}:{\tilde{D}}_{\Delta_{0}}\left(P_{Y},P_{0}\right)\leq \lambda }{\tilde{D}}_{\Delta_{1}}\left(P_{Y},P_{1}\right). \end{array}$$

In Equation (22), we made explicit the dependence on the parameter λ in the notation of the error exponent, since this is useful in the sequel.

The proof of Theorem 3, which appears in Appendix A.3, is based on Sanov’s theorem [29,30], by exploiting the compactness of the set $\left\{P_{Y}:{\tilde{D}}_{\Delta_{0}}\left(P_{Y},P_{0}\right)\leq \lambda \right\}$.

From Theorem 3 it follows that $\varepsilon_{\mathrm{FN}}\left(\lambda \right)=0$ whenever there exists a PMF $P_{Y}$ inside the set $\left\{P_{Y}:{\tilde{D}}_{\Delta_{0}}\left(P_{Y},P_{0}\right)\leq \lambda \right\}$ with $\Delta_{1}$-limited expected distortion from $P_{1}$. When this condition does not hold, $P_{\mathrm{FN}}\left(\Phi^{*},A_{\Delta_{1}}^{*}\right)\to 0$ exponentially rapidly.

For a partially active attacker, the error exponent in Equation (22) becomes

(23) $$\begin{array}{cc} \varepsilon_{\mathrm{FN}}\left(\lambda \right)= & \min_{P_{Y}:D\left(P_{Y},P_{0}\right)\leq \lambda }{\tilde{D}}_{\Delta_{1}}\left(P_{Y},P_{1}\right). \end{array}$$

It can be shown that the error exponent in Equation (23) is the same as the error exponent of Theorem 2 in [11] (and Theorem 2 in [31]), where deterministic strategies are considered for both the defender and the attacker. Such equivalence can be explained as follows. As already pointed, the optimal defense strategy in Equation (17) and the deterministic rule found in [11] are asymptotically equivalent (see the discussion immediately after Lemma 1). Concerning the attacker, even in the more general setup (with randomized strategies) considered here, an asymptotically optimal attack could be derived as in [11], that is, by considering the best response to the dominant defense strategy in [11]. Such attack consists of minimizing the divergence with respect to $P_{0}$, namely $D({\hat{P}}_{y}||P_{0})$, over all the admissible sequences y, and then is deterministic. Therefore, concerning the partially active case, the asymptotic behavior of the game is equivalent to the one in [11]. The main difference between the setup in [11] and the more general one addressed in this paper relies on the *kind* of game equilibrium, which is stronger here (namely, a *dominant* equilibrium) due to the existence of dominant strategies for both the defender and the attacker, rather than for the defender only.

When the distortion function *d* is a metric, we can state the following result, whose proof appears in Appendix A.4.

**Theorem** **4.** *When the distortion function d is a metric, Equation (22) can be rephrased as*

(24) $$\begin{array}{cc} \varepsilon_{\mathrm{FN}}\left(\lambda \right)= & \min_{P_{Y}:D\left(P_{Y}∥P_{0}\right)\leq \lambda }{\tilde{D}}_{\Delta_{0}+\Delta_{1}}\left(P_{Y},P_{1}\right). \end{array}$$

Comparing Equations (23) and (24) is insightful for understanding the difference between the fully active and partially active cases. Specifically, the FN error exponents of both cases are the same when the distortion under $H_{1}$ in the partially active case is $\Delta_{0}+\Delta_{1}$ (instead of $\Delta_{1}$).

When *d* is not a metric, Equation (24) is only an upper bound on $\varepsilon_{\mathrm{FN}}\left(\lambda \right)$, as can be seen from the proof of Theorem 4. Accordingly, in the general case (*d* is not a metric), applying distortion levels $\Delta_{0}$ and $\Delta_{1}$ to sequences from, respectively, $H_{0}$ and $H_{1}$ (in the fully active setup) is more favorable to the attacker with respect to applying a distortion $\Delta_{0}+\Delta_{1}$ to sequences from $H_{0}$ only (in the partially active setup).

## 5. The Bayesian Detection Game

In this section, we study the Bayesian game (Definition 2). In contrast to the Neyman–Pearson game, in the Bayesian game, the optimal defense strategy is found by assuming that the strategy played by the attacker, namely the optimal pair of channels $\left(A_{0}^{*},A_{1}^{*}\right)$ of Theorem 1, is known to the defender, that is, by exploiting the rationalizability argument (see Section 2.1). Accordingly, the resulting optimal strategy is not dominant, thus the associated equilibrium is weaker compared to that of the Neyman–Pearson game.

### 5.1. Optimal Defense and Game Equilibrium

Since the payoff in Equation (3) is a special case of Equation (5) with γ=1 and $\beta =e^{an}$, for any defense strategy $\Phi \in S_{D}$, the asymptotically optimal attack channels under $H_{0}$ and $H_{1}$ are given by Theorem 1, and correspond to the pair $\left(A_{\Delta_{0}}^{*},A_{\Delta_{1}}^{*}\right)$. Then, we can determine the best defense strategy by assuming that the attacker will play $\left(A_{\Delta_{0}}^{*},A_{\Delta_{1}}^{*}\right)$ and evaluating the best response of the defender to this pair of channels.

Our solution for the Bayesian detection game is given in the following theorem, whose proof appears in Appendix B.1.

**Theorem** **5.** Consider the Bayesian detection game of Definition 2. Let $Q_{0}^{*}\left(y\right)$ and $Q_{1}^{*}\left(y\right)$ be the probability distributions induced by channels $A_{\Delta_{0}}^{*}$ and $A_{\Delta_{1}}^{*}$, respectively.

*Then,*

(25) $$\Phi^{\#}\left(H_{1}|y\right)=U\left(\frac{1}{n}\log\frac{Q_{1}^{*}\left(y\right)}{Q_{0}^{*}\left(y\right)}-a\right)$$

*is the optimal defense strategy.*

*If, in addition, the distortion measure is additive, the defense strategy*

(26) $$\Phi^{†}\left(H_{1}|y\right)=U\left({\tilde{D}}_{\Delta_{0}}^{n}\left({\hat{P}}_{y},P_{0}\right)-{\tilde{D}}_{\Delta_{1}}^{n}\left({\hat{P}}_{y},P_{1}\right)-a\right)$$

*is asymptotically optimal.*

It is useful to provide the asymptotically optimal strategy, $\Phi^{†}$, in addition to the optimal one, $\Phi^{\#}$, for the following reason: while $\Phi^{\#}$ requires the non-trivial computation of the two probabilities $Q_{1}\left(y\right)$ and $Q_{0}\left(y\right)$, the strategy $\Phi^{†}$, which leads to the same payoff asymptotically, is easier to implement because of its single-letter form.

According to the asymptotically optimal defense strategy defined by the above theorem, given the observed sequence ***y***, the decision is made by comparing the value of the difference ${\tilde{D}}_{\Delta_{0}}^{n}\left({\hat{P}}_{y}|P_{0}\right)-{\tilde{D}}_{\Delta_{1}}^{n}\left({\hat{P}}_{y}|P_{1}\right)$ against the threshold. Then, the functions ${\tilde{D}}_{\Delta_{0}}^{n}$ and ${\tilde{D}}_{\Delta_{1}}^{n}$ take the role of the K-L divergence functions when the likelihood ratio test is performed in the non-adversarial case [19].

We now state the following theorem.

**Theorem** **6.** Consider the Bayesian game of Definition 2. Let $\left(A_{\Delta_{0}}^{*},A_{\Delta_{1}}^{*}\right)$ be the attack strategy of Theorem 1 and let $\Phi^{\#}$ and $\Phi^{†}$ be the defense strategies defined, respectively, in Equations (25) and (26). The profiles $\left(\Phi^{\#},\left(A_{\Delta_{0}}^{*},A_{\Delta_{1}}^{*}\right)\right)$ and $\left(\Phi^{†},\left(A_{\Delta_{0}}^{*},A_{\Delta_{1}}^{*}\right)\right)$ are asymptotic rationalizable equilibria of the game.

The analysis in this section can be easily generalized to any payoff function defined as in Equation (5), i.e., for any γ,β≥0.

Finally, we observe that the equilibrium found in the Bayesian case (namely, a rationalizable equilibrium) is weaker with respect to the equilibrium derived for the Neyman–Pearson game (namely, a dominant equilibrium) is a consequence of the fact that the Bayesian game is defined in a less restrictive manner than the Neyman–Pearson game. This is due to the conservative approach adopted in the latter: while in the Bayesian game the defender cares about both FP and FN probabilities and their tradeoff, in the Neymam–Pearson game the defender does not care about the value of the FP probability provided that its exponent is larger than λ, which is automatically guaranteed by restricting the set of strategies. This restriction simplifies the game so that a dominant strategy can be found for the restricted game.

### 5.2. Equilibrium Payoff

We now derive the equilibrium payoff of the Bayesian game. As in the Neyman–Pearson game, we assume an additive distortion measure. For simplicity, we focus on the asymptotically optimal defense strategy $\Phi^{†}$. We have the following theorem.

**Theorem** **7.** Let the Bayesian detection game be as in Definition 2. Let $\left(\Phi^{†},\left(A_{\Delta_{0}}^{*},A_{\Delta_{1}}^{*}\right)\right)$ be the equilibrium profile of Theorem 6. The asymptotic exponential rate of the equilibrium Bayes payoff u is given by

(27) $$\begin{array}{cc} -\lim_{n\to \infty } & \frac{1}{n}\ln\left(u(\Phi^{†},\left(A_{\Delta_{0}}^{*},A_{\Delta_{1}}^{*}\right))\right)=\\ & \min_{P_{Y}}\left(\max\left\{{\tilde{D}}_{\Delta_{1}}\left(P_{Y},P_{1}\right),\left({\tilde{D}}_{\Delta_{0}}\left(P_{Y},P_{0}\right)-a\right)\right\}\right). \end{array}$$

The proof appears in Appendix B.2.

According to Theorem 7, the asymptotic exponent of *u* is zero if there exists a PMF $P_{Y}^{*}$ with $\Delta_{1}$-limited expected distortion from $P_{1}$ such that ${\tilde{D}}_{\Delta_{0}}\left(P_{Y}^{*},P_{0}\right)\leq a$. Therefore, when we focus on the case of zero asymptotic exponent of the payoff, the parameter *a* plays a role similar to λ in the Neyman–Pearson game. By further inspecting the exponent expressions of Theorems 7 and 3, we observe that, when a=λ, the exponent in Equation (27) is smaller than or equal to the one in Equation (22), where equality holds only when both Equations (22) and (27) vanish. However, comparing these two cases in the general case is difficult because of the different definition of the payoff functions and, in particular, the different role taken by the parameters λ and *a*. In the Neyman–Pearson game, in fact, the payoff corresponds to the FN probability and is not affected by the value of the FP probability, provided that its exponent is larger than λ; in this way, the ratio between FP and FN error exponent at the equilibrium is generally smaller than λ (a part for the case in which the asymptotic exponent of the payoff is zero). In the Bayesian case, the payoff is a weighted combination of the two types of errors and then the term with the largest exponent is the dominating term, namely, the one which determines the asymptotic behavior; in this case, the parameter *a* determines the exact tradeoff between the FP and FN exponent in the equilibrium payoff.

## 6. Source Distinguishability

In this section, we investigate the performance of the Neyman–Pearson and Bayesian games as a function of λ and *a*, respectively. From the expressions of the equilibrium payoff exponents, it is clear that increase (and then the equilibrium payoffs of the games decrease) as λ and *a* decrease, respectively. In particular, by letting λ=0 and a=0, we obtain the largest achievable exponents of both games, which correspond to the best achievable performance for the defender. Therefore, we say that two sources are *distinguishable* under the Neyman–Pearson (respectively, Bayesian) setting, if there exists a value of λ (respectively, α) such that the FP and FN exponents at the equilibrium of the game are simultaneously strictly positive. When such a condition does not hold, we say that the sources are indistinguishable. Specifically, in this section, we characterize, under both the Neyman–Pearson and the Bayesian settings, the *indistinguishability region*, defined as the set of the alternative sources that cannot be distinguished from a given source $P_{0}$, given the attack distortion levels $\Delta_{0}$ and $\Delta_{1}$. Although each game has a different asymptotic behavior, we show that the indistinguishability regions in the Neyman–Pearson and the Bayesian settings are the same. The study of the distinguishability between the sources under adversarial conditions, performed in this section, in a way extends the Chernoff–Stein lemma [19] to the adversarial setup (see [31]).

We start by proving the following result for the Neyman–Pearson game.

**Theorem** **8.** *Given two memoryless sources $P_{0}$ and $P_{1}$ and distortion levels $\Delta_{0}$ and $\Delta_{1}$, the maximum achievable FN exponent for the Neyman–Pearson game is:*

(28) $$\begin{array}{c} \lim_{\lambda \to 0}\;\varepsilon_{\mathrm{FN}}\left(\lambda \right)\;=\varepsilon_{\mathrm{FN}}\left(0\right)=\min_{\left\{P_{Y|X}:E_{XY}d\left(X,Y\right)\leq \Delta_{0},\;{\left(P_{XY}\right)}_{X}=P_{0}\right\}}{\tilde{D}}_{\Delta_{1}}\left(P_{Y},P_{1}\right), \end{array}$$

*where $\varepsilon_{\mathrm{FN}}\left(\lambda \right)$ is as in Theorem 3.*

The theorem is an immediate consequence of the continuity of $\varepsilon_{\mathrm{FN}}\left(\lambda \right)$ as $\lambda \to 0^{+}$, which follows by the continuity of ${\tilde{D}}_{\Delta }$ with respect to $P_{Y}$ and the density of the set $\left\{P_{Y}:{\tilde{D}}_{\Delta_{0}}\left(P_{Y},P_{0}\right)\leq \lambda \right\}$ in $\left\{P_{Y}:{\tilde{D}}_{\Delta_{0}}\left(P_{Y},P_{0}\right)=0\right\}$ as $\lambda \to 0^{+}$ (It holds true from Property 1).

We notice that, if $\Delta_{0}=\Delta_{1}=0$, there is only an admissible point in the set in Equation (28), for which $P_{Y}=P_{0}$; then, $\varepsilon_{\mathrm{FN}}\left(0\right)=D(P_{0}\left|\right|P_{1})$, which corresponds to the best achievable FN exponent known from the classical literature for the non-adversarial case (Stein lemma [19] (Theorem 11.8.3)).

Regarding the Bayesian setting, we have the following theorem, the proof of which appears in Appendix C.1.

**Theorem** **9.** *Given two memoryless sources $P_{0}$ and $P_{1}$ and distortion levels $\Delta_{0}$ and $\Delta_{1}$, the maximum achievable exponent of the equilibrium Bayes payoff is*

(29) $$\begin{array}{cc} -\lim_{a\to 0}\lim_{n\to \infty } & \frac{1}{n}\ln\left(u(\Phi^{†},\left(A_{\Delta_{0}}^{*},A_{\Delta_{1}}^{*}\right))\right)=\\ & \min_{P_{Y}}\left(\max\left\{{\tilde{D}}_{\Delta_{1}}\left(P_{Y},P_{1}\right),{\tilde{D}}_{\Delta_{0}}\left(P_{Y},P_{0}\right)\right\}\right), \end{array}$$

*where the inner limit at the left hand side is as defined in Theorem 7.*

Since ${\tilde{D}}_{\Delta_{1}}\left(P_{Y},P_{1}\right)$, and similarly ${\tilde{D}}_{\Delta_{0}}\left(P_{Y},P_{0}\right)$, are convex functions of $P_{Y}$, and reach their minimum in $P_{1}$, respectively $P_{0}$ (the fact that ${\tilde{D}}_{\Delta_{0}}$ (${\tilde{D}}_{\Delta_{1}}$) is 0 in a $\Delta_{0}$-limited ($\Delta_{1}$-limited) neighborhood of $P_{0}$ ($P_{1}$), and not just in $P_{0}$ ($P_{1}$), does not affect the argument), the minimum over $P_{Y}$ of the maximum between these quantities (right-hand side of Equation (29)) is attained when ${\tilde{D}}_{\Delta_{1}}\left(P_{Y}^{*},P_{1}\right)={\tilde{D}}_{\Delta_{0}}\left(P_{Y}^{*},P_{0}\right)$, for some PMF $P_{Y}^{*}$. This resembles the best achievable exponent in the Bayesian probability of error for the non-adversarial case, which is attained when $D\left(P_{Y}^{*}∥P_{0}\right)=D\left(P_{Y}^{*}∥P_{1}\right)$ for some $P_{Y}^{*}$ (see [19] (Theorem 11.9.1)). In that case, from the expression of the divergence function, such $P_{Y}^{*}$ is found in a closed form and the resulting exponent is equivalent to the Chernoff information (see Section 11.9 [19]).

From Theorems 8 and 9, it follows that there is no positive λ, respectively *a*, for which the asymptotic exponent of the equilibrium payoff is strictly positive, if there exists a PMF $P_{Y}$ such that the following conditions are both satisfied:

(30) $$\left\{\begin{array}{c} {\tilde{D}}_{\Delta_{0}}\left(P_{Y},P_{0}\right)=0\\ {\tilde{D}}_{\Delta_{1}}\left(P_{Y},P_{1}\right)=0. \end{array}\right.$$

In this case, then, $P_{0}$ and $P_{1}$ are indistinguishable under both the Neyman–Pearson and the Bayesian settings. We observe that the condition ${\tilde{D}}_{\Delta }\left(P_{Y},P_{X}\right)=0$ is equivalent to the following:

(31) $$\min_{Q_{XY}:\begin{array}{c} {\left(Q_{XY}\right)}_{X}=P_{X}\\ {\left(Q_{XY}\right)}_{Y}=P_{Y} \end{array}}E_{XY}d\left(X,Y\right)\leq \Delta ,$$

where the expectation $E_{XY}$ is with respect to $Q_{XY}$. For ease of notation, in Equation (31), we used ${\left(Q_{XY}\right)}_{Y}=P_{Y}$ (respectively ${\left(Q_{XY}\right)}_{X}=P_{X}$) as short for $\sum_{x}Q_{XY}\left(x,y\right)=P_{Y}\left(y\right)$, ∀y∈A (respectively $\sum_{y}Q_{XY}\left(x,y\right)=P_{X}\left(x\right)$, ∀x∈A), where $Q_{XY}$ is a joint PMF and $P_{X}$ and $P_{Y}$ are its marginal PMFs.

In computer vision applications, the left-hand side of Equation (31) is known as the *Earth Mover Distance* (EMD) between $P_{X}$ and $P_{Y}$, which is denoted by ${EMD}_{d}\left(P_{X},P_{Y}\right)$ (or, by symmetry, ${EMD}_{d}\left(P_{Y},P_{X}\right)$) [32]. It is also known as the ρ-bar distortion measure [33].

A brief comment concerning the analogy between the minimization in Equation (31) and *optimal transport theory* is worth expounding. The minimization problem in Equation (31) is known in the Operations Research literature as *Hitchcock Transportation Problem* (TP) [34]. Referring to the original Monge formulation of this problem [35], $P_{X}$ and $P_{Y}$ can be interpreted as two different ways of piling up a certain amount of soil; then, $P_{XY}\left(x,y\right)$ denotes the quantity of soil shipped from location (source) *x* in $P_{X}$ to location (sink) *y* in $P_{Y}$ and d(x,y) is the cost for shipping a unitary amount of soil from *x* to *y*. In transport theory terminology, $P_{XY}$ is referred to as *transportation map*. According to this perspective, evaluating the *EMD* corresponds to finding the minimal transportation cost of moving a pile of soil into the other. Further insights on this parallel can be found in [31].

We summarize our findings in the following corollary, which characterizes the conditions for distinguishability under both the Neyman–Pearson and the Bayesian setting.

**Corollary** **1** (Corollary to Theorems 8 and 9)**.** *Given a memoryless source $P_{0}$ and distortion levels $\Delta_{0}$ and $\Delta_{1}$, the set of the PMFs that cannot be distinguished from $P_{0}$ in both the Neyman–Pearson and Bayesian settings is given by*

(32) $$\Gamma =\left\{P:\min_{P_{Y}:{EMD}_{d}\left(P_{Y},P_{0}\right)\leq \Delta_{0}}{EMD}_{d}\left(P_{Y},P\right)\leq \Delta_{1}\right\}.$$

Set Γ is the indistinguishability region. By definition (see the beginning of this section), the PMFs inside Γ are those for which, as a consequence of the attack, the FP and FN probabilities cannot go to zero simultaneously with strictly positive exponents. Clearly, if $\Delta_{0}=\Delta_{1}=0$, that is, in the non-adversarial case, $\Gamma =\{P_{0}\}$, as any two distinct sources are always distinguishable.

When *d* is a metric, for a given P∈Γ, the computation of the optimal $P_{Y}$ can be traced back to the computation of the *EMD* between $P_{0}$ and *P*, as stated by the following corollary, whose proof appears in Appendix C.2.

**Corollary** **2** (Corollary to Theorems 8 and 9)**.** *When d is a metric, given the source $P_{0}$ and distortion levels $\Delta_{0}$ and $\Delta_{1}$, for any fixed P, the minimum in Equation (32) is achieved when*

(33) $$P_{Y}=\alpha P_{0}+\left(1-\alpha \right)P,\;\alpha =1-\frac{\Delta_{0}}{EMD(P_{0},P)}.$$

*Then, the set of PMFs that cannot be distinguished from $P_{0}$ in the Neyman–Pearson and Bayesian setting is given by*

(34) $$\Gamma =\{P:{EMD}_{d}\left(P_{0},P\right)\leq \Delta_{0}+\Delta_{1}\}.$$

According to Corollary 2, when *d* is a metric, the performance of the game depends only on the sum of distortions, $\Delta_{0}+\Delta_{1}$, and it is immaterial how this amount is distributed between the two hypotheses.

In the general case (*d* not a metric), the condition on the *EMD* stated in Equation (34) is sufficient in order for $P_{0}$ and *P* be indistinguishable, that is $\Gamma ⊇\{P:{EMD}_{d}\left(P_{0},P\right)\leq \Delta_{0}+\Delta_{1}\}$ (see discussion in Appendix C.2, at the end of the proof of Corollary 2).

Furthermore, in the case of an $L_{p}^{p}$ distortion function (p≥1), i.e., $d\left(x,y\right)=\sum_{i=1}^{n}{\left|x_{i}-y_{i}\right|}^{p}$, we have the following corollary.

**Corollary** **3** (Corollary to Theorems 8 and 9)**.** *When d is the $L_{p}^{p}$ distortion function, for some p≥1, the set Γ can be bounded as follows*

(35) $$\Gamma \subseteq \{P:{EMD}_{L_{p}^{p}}\left(P_{0},P\right)\leq {\left({\Delta_{0}}^{1/p}+{\Delta_{1}}^{1/p}\right)}^{p}\}.$$

Corollary 3 can be proven by exploiting the Hölder inequality [36] (see Appendix C.3).

## 7. Concluding Remarks

We consider the problem of binary hypothesis testing when an attacker is active under both hypotheses, and then an attack is carried out aiming at both false negative and false positive errors. By modeling the defender–attacker interaction as a game, we defined and solved two different detection games: the Neyman–Pearson and the Bayesian game. This paper extends the analysis in [11], where the attacker is active under the alternative hypothesis only. Another aspect of greater generality is that here both players are allowed to use randomized strategies. By relying on the method of types, the main result of this paper is the existence of an attack strategy, which is both *dominant* and *universal*, that is, optimal regardless of the statistics of the sources. The optimal attack strategy is also independent of the underlying hypothesis, namely *fully-universal*, when the distortion introduced by the attacker in the two cases is the same. From the analysis of the asymptotic behavior of the equilibrium payoff, we are able to establish conditions under which the sources can be reliably distinguished in the fully active adversarial setup. The theory developed permits to assess the security of the detection in adversarial setting and give insights on how the detector should be designed in such a way to make the attack hard.

Among the possible directions for future work, we mention the extension to continuous alphabets, which calls for an extension of the method of types to this case, or to more realistic models of finite alphabet sources, still amenable to analysis, such as Markov sources. As mentioned in the Introduction, it would be also relevant to extend the analysis to higher order statistics. In fact, the techniques introduced in this paper are very general and lend themselves to extensions that allow richer sets of sufficient statistics (as currently pursued in an ongoing work of the first two coauthors). This generalization, however, comes at the price of an increased complexity of the analysis with regard to both the expressions of the equilibrium strategies and the performance achieved by the two parties at the equilibrium. More specifically, given the set of statistics used by the defender, it turns out that the optimal attacker also uses the very same statistics (plus the empirical correlation with the source sequence, due to the distortion constraint) and the optimal attack channel is given in terms of uniform distributions across conditional types that depend on these set of statistics. Therefore, in a sequence of games, where the defender exploits more and more statistics, so would the attacker, and it would be interesting to explore the limiting behaviour of this process.

We also mention the case of unknown sources, where the source statistics are estimated from training data, possibly corrupted by the attacker. In this scenario, the detection game has been studied for a partially active case, with both uncorrupted and corrupted training data [13,14]. The extension of such analyses to the fully active scenario represents a further interesting direction for future research.

Finally, we stress that, in this paper, as well as in virtually all prior works using game theory to model the interplay between the attacker and the defender, it is assumed that the attacker is always present. This is a pessimistic or worst-case assumption, leading to the adoption of an overly conservative defense strategy (when the attacker is not present, in fact, better performance could be in principle achieved by adopting a non-adversarial detection strategy). A more far-reaching extension would require that the ubiquitous presence of the attacker is reconsidered, for instance by resorting to a two-steps analysis, where the presence or absence of the attacker is established in the first step, or by defining and studying more complex game models, e.g., games with incomplete information [37].

## Appendix A. Neyman–Pearson Detection Game

This appendix contains the proofs of the results in Section 4.

### Appendix A.1. Proof of Lemma 1

Whenever existent, the dominant defense strategy can be obtained by solving:

(A1) $$\min_{\Phi \in S_{D}}P_{\mathrm{FN}}\left(\Phi ,A_{1}\right),$$

for any attack channel $A_{1}$. Below, we first show that $P_{\mathrm{FN}}\left(\Phi^{*},A_{1}\right)\overset{\cdot }{\leq }P_{\mathrm{FN}}\left(\Phi ,A_{1}\right)$ for every $\Phi \in S_{D}$ and for every $A_{1}$, that is, $\Phi^{*}$ is asymptotically dominant. Then, by proving that $\max_{A\in C_{\Delta_{0}}}P_{\mathrm{FP}}\left(\Phi^{*},A\right)$ fulfills the FP constraint, we show that $\Phi^{*}$ is also admissible. Therefore, we can conclude that $\Phi^{*}\left(\cdot |y\right)$ asymptotically solves Equation (A1). Exploiting the memorylessness of $P_{0}$ and the permutation invariance of $\Phi (H_{1}|y)$ and d(x,y), for every $y^{\prime }\in A^{n}$ we have,

(A2) $$\begin{array}{ccc} e^{-\lambda n} & \geq & \max_{A}\sum_{x,y}P_{0}\left(x\right)A\left(y|x\right)\Phi \left(H_{1}|y\right)\\ & \geq & \sum_{y}\left(\sum_{x}P_{0}\left(x\right)A_{\Delta_{0}}^{*}\left(y|x\right)\right)\Phi \left(H_{1}|y\right)\\ & = & \sum_{y}\left(\sum_{x:d\left(x,y\right)\leq n\Delta_{0}}P_{0}\left(x\right)\cdot \frac{c_{n}\left(x\right)}{\left|T(y|x)\right|}\right)\Phi \left(H_{1}|y\right)\\ & \geq & {\left(n+1\right)}^{-|A|\cdot (|A|-1)}\sum_{y}\left(\sum_{x:d\left(x,y\right)\leq n\Delta_{0}}\cdot \frac{P_{0}\left(x\right)}{\left|T(y|x)\right|}\right)\Phi \left(H_{1}|y\right)\\ & \overset{\left(a\right)}{\geq } & {\left(n+1\right)}^{-|A|\cdot (|A|-1)}\left|T\left(y^{\prime }\right)\right|\left(\max_{x:d\left(x,y^{\prime }\right)\leq n\Delta_{0}}\left|T\left(x|y^{\prime }\right)\right|\cdot \frac{P_{0}\left(x\right)}{\left|T(y^{\prime }|x)\right|}\right)\Phi \left(H_{1}|y^{\prime }\right)\\ & \overset{\left(b\right)}{=} & {\left(n+1\right)}^{-|A|\cdot (|A|-1)}\Phi \left(H_{1}|y^{\prime }\right)\max_{x:d\left(x,y^{\prime }\right)\leq n\Delta_{0}}P_{0}\left(x\right)\cdot \left|T\left(x\right)\right|\\ & \geq & \Phi \left(H_{1}|y^{\prime }\right)\max_{x:d\left(x,y^{\prime }\right)\leq n\Delta_{0}}\frac{e^{-nD({\hat{P}}_{x}∥P_{0})}}{{\left(n+1\right)}^{{\left|A\right|}^{2}\cdot \left(|A|-1\right)}}\\ & = & \Phi \left(H_{1}|y^{\prime }\right)\frac{\exp\left\{-n\min_{x:d\left(x,y^{\prime }\right)\leq n\Delta_{0}}D\left({\hat{P}}_{x}∥P_{0}\right)\right\}}{{\left(n+1\right)}^{{\left|A\right|}^{2}\cdot \left(|A|-1\right)}}, \end{array}$$

where (a) is due to the permutation invariance of the distortion function *d* and (b) is due to the identity |T(x)|·|T(y|x)|≡|T(y)|·|T(x|y)|≡|T(x,y)|.

It now follows that

(A3) $$\Phi \left(H_{1}|y\right)\overset{\cdot }{\leq }\exp\left\{-n\left[\lambda -\min_{x:d\left(x,y\right)\leq n\Delta_{0}}D\left({\hat{P}}_{x}∥P_{0}\right)\right]\right\}.$$

Since $\Phi (H_{1}|y)$ is a probability,

(A4) $$\begin{array}{cc} \Phi (H_{1}|y) & \overset{\cdot }{\leq }\min\left\{1,\exp\left[-n\left(\lambda -\min_{x:d\left(x,y\right)\leq n\Delta_{0}}D\left({\hat{P}}_{x}∥P_{0}\right)\right)\right]\right\}\\ & =\Phi^{*}\left(H_{1}|y\right). \end{array}$$

Consequently, $\Phi^{*}\left(H_{0}|y\right)\overset{\cdot }{\leq }\Phi \left(H_{0}|y\right)$ for every ***y***, and so, $P_{\mathrm{FN}}\left(\Phi^{*},A_{1}\right)\overset{\cdot }{\leq }P_{\mathrm{FN}}\left(\Phi ,A_{1}\right)$ for every $A_{1}$. For convenience, let us denote

$$k_{n}\left(y\right)=\lambda -\min_{x:d\left(x,y\right)\leq n\Delta_{0}}D\left({\hat{P}}_{x}∥P_{0}\right),$$

so that $\Phi^{*}\left(H_{1}|y\right)=\min\left\{1,e^{-n\cdot k_{n}\left(y\right)}\right\}$. We now show that $\Phi^{*}\left(H_{1}|y\right)$ satisfies the FP constraint, up to a polynomial term in *n*, i.e., it satisfies the constraint asymptotically.

(A5) $$\begin{array}{cc} \max_{A\in C_{\Delta_{0}}}P_{\mathrm{FP}}\left(\Phi^{*},A\right) & \leq {\left(n+1\right)}^{\left|A|\cdot (|A|-1\right)}P_{\mathrm{FP}}\left(\Phi^{*},A^{*}\right)\\ & ={\left(n+1\right)}^{\left|A|\cdot (|A|-1\right)}\sum_{x,y}P_{0}\left(x\right)A_{\Delta_{0}}^{*}\left(y|x\right)\Phi^{*}\left(H_{1}|y\right)\\ & ={\left(n+1\right)}^{\left|A|\cdot (|A|-1\right)}\sum_{\left(x,y\right):d\left(x,y\right)\leq n\Delta_{0}}P_{0}\left(x\right)\cdot \frac{c_{n}\left(x\right)}{\left|T(y|x)\right|}\cdot \Phi^{*}\left(H_{1}|y\right)\\ & \leq {\left(n+1\right)}^{\left|A|\cdot (|A|-1\right)}\sum_{\left(x,y\right):d\left(x,y\right)\leq n\Delta_{0}}\frac{P_{0}\left(x\right)}{\left|T(y|x)\right|}\cdot \Phi^{*}\left(H_{1}|y\right)\\ & \leq {\left(n+1\right)}^{2|A|\cdot (|A|-1)}\sum_{y}\left(\max_{x:d\left(x,y\right)\leq n\Delta_{0}}\left|T\left(x|y\right)\right|\cdot \frac{P_{0}\left(x\right)}{\left|T(y|x)\right|}\right)\Phi^{*}\left(H_{1}|y\right)\\ & ={\left(n+1\right)}^{2|A|\cdot (|A|-1)}\left(\sum_{{\hat{P}}_{y}:k_{n}\left(y\right)\geq 0}e^{-nk_{n}\left(y\right)}\left(\max_{x:d\left(x,y\right)\leq n\Delta_{0}}\left|T\left(x\right)\right|\cdot P_{0}\left(x\right)\right)+\right.\\ & \;\left.+\sum_{{\hat{P}}_{y}:k_{n}\left(y\right)<0}\left(\max_{x:d\left(x,y\right)\leq n\Delta_{0}}\left|T\left(x\right)\right|\cdot P_{0}\left(x\right)\right)\right)\\ & \leq {\left(n+1\right)}^{2|A|\cdot (|A|-1)}\left(\sum_{{\hat{P}}_{y}:k_{n}\left(y\right)\geq 0}e^{-n\lambda }+\right.\\ & \;\left.+\sum_{{\hat{P}}_{y}:k_{n}\left(y\right)<0}\exp\left\{-n\min_{x:d\left(x,y\right)\leq n\Delta_{0}}D\left({\hat{P}}_{x}∥P_{0}\right)\right\}\right)\\ & \leq {\left(n+1\right)}^{\left|A|(|A{|}^{2}+|A|-1\right)}e^{-n\lambda }. \end{array}$$

### Appendix A.2. Proof of Property 1

We next prove that for any two PMFs $P_{Y_{1}}$ and $P_{Y_{2}}$ and any λ∈(0,1),

(A6) $${\tilde{D}}_{\Delta }\left(\lambda P_{Y_{1}}+\left(1-\lambda \right)P_{Y_{2}},P\right)\leq \lambda {\tilde{D}}_{\Delta }\left(P_{Y_{1}},P\right)+\left(1-\lambda \right){\tilde{D}}_{\Delta }\left(P_{Y_{2}},P\right).$$

Let us rewrite ${\tilde{D}}_{\Delta }$ in Equation (21) by expressing the minimization in terms of the joint PMF $P_{XY}$:

(A7) $${\tilde{D}}_{\Delta }\left(P_{Y},P\right)\overset{▵}{=}\min_{\left\{Q_{XY}:E_{XY}d\left(X,Y\right)\leq \Delta ,{\left(Q_{XY}\right)}_{Y}=P_{Y}\right\}}D\left({\left(Q_{XY}\right)}_{X}∥P\right),$$

where we used ${\left(Q_{XY}\right)}_{Y}=P_{Y}$ as short for $\sum_{x}Q_{XY}\left(x,y\right)=P_{Y}\left(y\right)$, ∀y, and we made explicit the dependence of $D(P_{X}∥P)$ on $Q_{XY}$. Accordingly:

(A8) $${\tilde{D}}_{\Delta }\left(\lambda P_{Y_{1}}+\left(1-\lambda \right)P_{Y_{2}},P\right)=\min_{\left\{Q_{XY}:E_{XY}d\left(X,Y\right)\leq \Delta ,\;{\left(Q_{XY}\right)}_{Y}=\lambda P_{Y_{1}}+\left(1-\lambda \right)P_{Y_{2}}\right\}}D\left({\left(Q_{XY}\right)}_{X}∥P\right).$$

We find it convenient to rewrite the right-hand side of Equation (A8) by minimizing over pairs of PMFs $\left(Q_{XY}^{\prime },Q_{XY}^{\prime \prime }\right)$ and considering the convex combination of these PMFs with weights λ and (1−λ), in place of $Q_{XY}$; hence,

(A9) $${\tilde{D}}_{\Delta }\left(\lambda P_{Y_{1}}+\left(1-\lambda \right)P_{Y_{2}},P\right)=\min_{(Q_{XY}^{\prime },Q_{XY}^{\prime \prime })\in H}D\left(\lambda {\left(Q_{XY}^{\prime }\right)}_{X}+\left(1-\lambda \right){\left(Q_{XY}^{\prime \prime }\right)}_{X}∥P\right),$$

where

(A10) $$\begin{array}{cc} H= & \left\{\left(Q_{XY}^{\prime },Q_{XY}^{\prime \prime }\right):\lambda {\left(Q_{XY}^{\prime }\right)}_{Y}+\left(1-\lambda \right){\left(Q_{XY}^{\prime \prime }\right)}_{Y}=\lambda P_{Y_{1}}+\left(1-\lambda \right)P_{Y_{2}},\right.\\ & \;\left.\lambda E_{XY}^{\prime }d\left(X,Y\right)+\left(1-\lambda \right)E_{XY}^{\prime \prime }d\left(X,Y\right)\leq \Delta \right\}. \end{array}$$

Let

(A11) $$\begin{array}{cc} H^{\prime }= & \left\{Q_{XY}^{\prime }:E_{XY}^{\prime \prime }d\left(X,Y\right)\leq \Delta ,{\left(Q_{XY}^{\prime }\right)}_{Y}=P_{Y_{1}}\right\}\times \left\{Q_{XY}^{\prime \prime }:E_{XY}^{\prime \prime }d\left(X,Y\right)\leq \Delta ,{\left(Q_{XY}^{\prime \prime }\right)}_{Y}=P_{Y_{2}}\right\}; \end{array}$$

then, $H^{\prime }\subset H$, where the set $H^{\prime }$ is separable in $Q_{XY}^{\prime }$ and $Q_{XY}^{\prime }$. Accordingly, Equations (A9) and (A10) can be upper bounded by

(A12) $$\min_{Q_{XY}^{\prime }:E_{XY}^{\prime \prime }d\left(X,Y\right)\leq \Delta ,{\left(Q_{XY}^{\prime }\right)}_{Y}=P_{Y_{1}}}\;\min_{Q_{XY}^{\prime \prime }:E_{XY}^{\prime \prime }d\left(X,Y\right)\leq \Delta ,{\left(Q_{XY}^{\prime \prime }\right)}_{Y}=P_{Y_{2}}}D\left(\lambda {\left(Q_{XY}^{\prime }\right)}_{X}+\left(1-\lambda \right){\left(Q_{XY}^{\prime \prime }\right)}_{X}∥P\right).$$

By the convexity of $D\left({\left(Q_{XY}\right)}_{X}∥P\right)$ with respect to $Q_{XY}$ (this is a consequence of the fact that the divergence function is convex in its arguments and the operation ${\left(\cdot \right)}_{X}$ is linear (see Theorem 2.7.2, [19])), it follows that

(A13) $$D\left(\lambda {\left(Q_{XY}^{\prime }\right)}_{X}+\left(1-\lambda \right){\left(Q_{XY}^{\prime \prime }\right)}_{X}∥P\right)\leq \lambda D\left({\left(Q_{XY}^{\prime }\right)}_{X}∥P\right)+\left(1-\lambda \right)D\left({\left(Q_{XY}^{\prime \prime }\right)}_{X}∥P\right).$$

Note that the above relation is not strict since it might be that ${\left(Q_{XY}^{\prime }\right)}_{X}={\left(Q_{XY}^{\prime \prime }\right)}_{X}=P$. Then, an upper bound for ${\tilde{D}}_{\Delta }\left(\lambda P_{Y_{1}}+\left(1-\lambda \right)P_{Y_{2}},P\right)$ is given by

(A14) $$\min_{Q_{XY}^{\prime }:\sum_{x}Q_{XY}^{\prime }=P_{Y_{1}},E_{XY}^{\prime \prime }d\left(X,Y\right)\leq \Delta }\lambda D\left({\left(Q_{XY}^{\prime }\right)}_{X}∥P\right)+\min_{Q_{XY}^{\prime \prime }:{\left(Q_{XY}^{\prime \prime }\right)}_{Y}=P_{Y_{2}},E_{XY}^{\prime \prime }d\left(X,Y\right)\leq \Delta }\left(1-\lambda \right)D\left({\left(Q_{XY}^{\prime \prime }\right)}_{X}∥P\right),$$

thus proving Equation (A6).

### Appendix A.3. Proof of Theorem 3

We start by proving the upper bound for the FN probability:

(A15) $$\begin{array}{ccc} P_{\mathrm{FN}}\left(\Phi^{*},A_{\Delta_{1}}^{*}\right) & = & \sum_{x,y}P_{1}\left(x\right)A_{\Delta_{1}}^{*}\left(y|x\right)\Phi^{*}\left(H_{0}|y\right)\\ & = & \sum_{y}\sum_{x:d\left(x,y\right)\leq n\Delta_{1}}P_{1}\left(x\right)\frac{c_{n}\left(x\right)}{\left|T(y|x)\right|}\left(1-e^{-n{\left[\lambda -{\tilde{D}}_{\Delta_{0}}^{n}\left({\hat{P}}_{y},P_{0}\right)\right]}_{+}}\right)\\ & \leq & \sum_{y}\sum_{x:d\left(x,y\right)\leq n\Delta_{1}}\frac{P_{1}\left(x\right)}{\left|T(y|x)\right|}\left(1-e^{-n{\left[\lambda -{\tilde{D}}_{\Delta_{0}}^{n}\left({\hat{P}}_{y},P_{0}\right)\right]}_{+}}\right)\\ & = & \sum_{y}\sum_{{\hat{P}}_{x|y}:E_{xy}d\left(X,Y\right)\leq \Delta_{1}}\left|T\left({\hat{P}}_{x|y}\right)\right|\frac{e^{-n[{\hat{H}}_{x}\left(X\right)+D\left({\hat{P}}_{x}∥P_{1}\right)]}}{\left|T({\hat{P}}_{y|x})\right|}\left(1-e^{-n{\left[\lambda -{\tilde{D}}_{\Delta_{0}}^{n}\left({\hat{P}}_{y},P_{0}\right)\right]}_{+}}\right)\\ & = & \sum_{{\hat{P}}_{y}}\sum_{{\hat{P}}_{x|y}:E_{xy}d\left(X,Y\right)\leq \Delta_{1}}\left|T\left({\hat{P}}_{x}\right)\right|e^{-n[{\hat{H}}_{x}\left(X\right)+D\left({\hat{P}}_{x}∥P_{1}\right)]}\left(1-e^{-n{\left[\lambda -{\tilde{D}}_{\Delta_{0}}^{n}\left({\hat{P}}_{y},P_{0}\right)\right]}_{+}}\right)\\ & = & \sum_{{\hat{P}}_{y}}\sum_{{\hat{P}}_{x|y}:E_{xy}d\left(X,Y\right)\leq \Delta_{1}}e^{-nD({\hat{P}}_{x}∥P_{1})}\left(1-e^{-n{\left[\lambda -{\tilde{D}}_{\Delta_{0}}^{n}\left({\hat{P}}_{y},P_{0}\right)\right]}_{+}}\right)\\ & = & \sum_{{\hat{P}}_{y}:{\tilde{D}}_{\Delta_{0}}^{n}\left({\hat{P}}_{y},P_{0}\right)<\lambda }\sum_{{\hat{P}}_{x|y}:E_{xy}d\left(X,Y\right)\leq \Delta_{1}}e^{-nD({\hat{P}}_{x}∥P_{1})}\left(1-e^{-n(\lambda -{\tilde{D}}_{\Delta_{0}}^{n}\left({\hat{P}}_{y},P_{0}\right))}\right)\\ & \leq & \sum_{{\hat{P}}_{y}:{\tilde{D}}_{\Delta_{0}}^{n}\left({\hat{P}}_{y},P_{0}\right)<\lambda }\sum_{{\hat{P}}_{x|y}:E_{xy}d\left(X,Y\right)\leq \Delta_{1}}e^{-nD({\hat{P}}_{x}∥P_{1})}\\ & \leq & {\left(n+1\right)}^{2|A|\cdot (|A|-1)}\exp\left\{-n\min_{{\hat{P}}_{y}:{\tilde{D}}_{\Delta_{0}}^{n}\left({\hat{P}}_{y},P_{0}\right)<\lambda }\left[\min_{{\hat{P}}_{x|y}:E_{xy}d\left(X,Y\right)\leq \Delta_{1}}D\left({\hat{P}}_{x}∥P_{1}\right)\right]\right\}\\ & \leq & {\left(n+1\right)}^{2|A|\cdot (|A|-1)}\exp\left\{-n\underset{P_{Y}:{\tilde{D}}_{\Delta_{0}}\left(P_{Y},P_{0}\right)<\lambda }{\inf}\left[\min_{P_{X|Y}:E_{XY}d\left(X,Y\right)\leq \Delta_{1}}D\left(P_{X}∥P_{1}\right)\right]\right\}\\ & \leq & {\left(n+1\right)}^{2|A|\cdot (|A|-1)}\exp\left\{-n\min_{P_{Y}:{\tilde{D}}_{\Delta_{0}}\left(P_{Y},P_{0}\right)\leq \lambda }\left[\min_{P_{X|Y}:E_{XY}d\left(X,Y\right)\leq \Delta_{1}}D\left(P_{X}∥P_{1}\right)\right]\right\}. \end{array}$$

Then:

(A16) $$\underset{n\to \infty }{lim sup}\frac{1}{n}\ln P_{\mathrm{FN}}\left(\Phi^{*},A_{\Delta_{1}}^{*}\right)\leq -\min_{P_{Y}:{\tilde{D}}_{\Delta_{0}}\left(P_{Y},P_{0}\right)\leq \lambda }\left[\min_{P_{X|Y}:E_{XY}d\left(X,Y\right)\leq \Delta_{1}}D\left(P_{X}∥P_{1}\right)\right].$$

We now move on to the lower bound.

(A17) $$\begin{array}{ccc} P_{\mathrm{FN}}\left(\Phi^{*},A_{\Delta_{1}}^{*}\right) & = & \sum_{x,y}P_{1}\left(x\right)A_{\Delta_{1}}^{*}\left(y|x\right)\Phi^{*}\left(H_{1}|y\right)\\ & \geq & {\left(n+1\right)}^{-|A|\cdot (|A|-1)}\sum_{y}\sum_{x:d\left(x,y\right)\leq n\Delta_{1}}\frac{P_{1}\left(x\right)}{\left|T(y|x)\right|}\left(1-e^{-n{\left[\lambda -{\tilde{D}}_{\Delta_{0}}^{n}\left({\hat{P}}_{y},P_{0}\right)\right]}_{+}}\right)\\ & = & {\left(n+1\right)}^{-|A|\cdot (|A|-1)}\sum_{{\hat{P}}_{y}:{\tilde{D}}_{\Delta_{0}}^{n}\left({\hat{P}}_{y},P_{0}\right)<\lambda }\sum_{{\hat{P}}_{x|y}:E_{xy}d\left(X,Y\right)\leq \Delta_{1}}e^{-nD({\hat{P}}_{x}∥P_{1})}\left(1-e^{-n(\lambda -{\tilde{D}}_{\Delta_{0}}^{n}\left({\hat{P}}_{y},P_{0}\right))}\right)\\ & \geq & {\left(n+1\right)}^{-|A|\cdot (|A|-1)}e^{-nD({\hat{P}}_{x}∥P_{1})}\left(1-e^{-n(\lambda -{\tilde{D}}_{\Delta_{0}}^{n}\left({\hat{P}}_{y},P_{0}\right))}\right), \end{array}$$

where, for a fixed *n*, ${\hat{P}}_{y}$ is a PMF that satisfies ${\tilde{D}}_{\Delta_{0}}^{n}\left({\hat{P}}_{y},P_{0}\right)\leq \lambda -\left(\ln n\right)/n$ and ${\hat{P}}_{x|y}$ is such that the distortion constraint is satisfied. Since the set of rational PMFs is dense in the probability simplex, two such sequences can be chosen in such a way that $\left({\hat{P}}_{y},{\hat{P}}_{x|y}\right)\to \left(P_{Y}^{*},P_{X|Y}^{*}\right)$ (we are implicitly exploiting the fact that set $\left\{{\tilde{D}}_{\Delta_{0}}^{n}\left({\hat{P}}_{y},P_{0}\right)<\lambda \right\}$ is dense in $\left\{{\tilde{D}}_{\Delta_{0}}\left(P_{Y},P_{0}\right)\leq \lambda \right\}$, for every λ>0, which holds true from Property 1), where

(A18) $$\left(P_{Y}^{*},P_{X|Y}^{*}\right)=\underset{\left(P_{Y},P_{X|Y}\right)}{\arg\min}\;\min_{P_{Y}:{\tilde{D}}_{\Delta_{0}}\left(P_{Y},P_{0}\right)\leq \lambda }\left[\min_{P_{X|Y}:E_{XY}d\left(X,Y\right)\leq \Delta_{1}}D\left(P_{X}∥P_{1}\right)\right].$$

Therefore, we can assert that:

(A19) $$\begin{array}{ccc} \underset{n\to \infty }{lim inf}\frac{1}{n}\ln P_{\mathrm{FN}}\left(\Phi^{*},A_{\Delta_{1}}^{*}\right) & \geq & \lim_{n\to \infty }\frac{1}{n}\ln\left[e^{-nD({\hat{P}}_{x}∥P_{1})}\left(1-e^{-n(\lambda -{\tilde{D}}_{\Delta_{0}}^{n}\left({\hat{P}}_{y},P_{0}\right))}\right)\right]\\ & = & -\lim_{n\to \infty }D\left({\hat{P}}_{x}∥P_{1}\right)\\ & = & -D(P_{X}^{*}∥P_{1})\\ & = & -\min_{P_{Y}:{\tilde{D}}_{\Delta_{0}}\left(P_{Y}∥P_{0}\right)\leq \lambda }\left[\min_{P_{X|Y}:E_{XY}d\left(X,Y\right)\leq \Delta_{1}}D\left(P_{X}∥P_{1}\right)\right]. \end{array}$$

By combining the upper and lower bounds, we conclude that limsup and liminf coincide. Therefore, the limit of the sequence $1/n\ln P_{\mathrm{FN}}$ exists and the theorem is proven.

### Appendix A.4. Proof of Theorem 4

First, observe that, by exploiting the definition of ${\tilde{D}}_{\Delta }$, Equation (22) can be rewritten as

(A20) $$\begin{array}{ccc} \varepsilon_{\mathrm{FN}} & = & \min_{P_{Y}:{\tilde{D}}_{\Delta_{0}}\left(P_{Y},P_{0}\right)\leq \lambda }\left(\min_{P_{X|Y}:E_{XY}d\left(X,Y\right)\leq \Delta_{1}}D\left(P_{X}∥P_{1}\right)\right)\\ & = & \min_{P_{Z}:D\left(P_{Z}∥P_{0}\right)\leq \lambda }\;\min_{P_{Y|Z}:E_{YZ}d\left(Y,Z\right)\leq \Delta_{0}}\left(\min_{P_{X|Y}:E_{XY}d\left(X,Y\right)\leq \Delta_{1}}D\left(P_{X}∥P_{1}\right)\right). \end{array}$$

To prove the theorem, we now show that Equation (A20) can be simplified as follows:

(A21) $$\begin{array}{cc} \varepsilon_{\mathrm{FN}}= & \min_{P_{Z}:D\left(P_{Z}∥P_{0}\right)\leq \lambda }\left(\min_{P_{X|Z}:E_{XZ}d\left(X,Z\right)\leq \Delta_{0}+\Delta_{1}}D\left(P_{X}∥P_{1}\right)\right), \end{array}$$

which is equivalent to Equation (24) (see Section 3). The equivalence of the expressions in Equations (A20) and (A21) follows from the equivalence of the two feasible sets for the PMF $P_{X}$. We first show that any feasible $P_{X}$ in Equation (A20) is also feasible in Equation (A21). Let $P_{X}$ be a feasible PMF in Equation (A20). By exploiting the properties of the triangular inequality property of the distance, we have that, regardless of the specific choice of the distributions $P_{Y|Z}$ and $P_{X|Y}$ in Equation (A20),

(A22) $$E_{XZ}d\left(X,Z\right)\leq E_{XYZ}\left[d\left(X,Y\right)+d\left(Y,Z\right)\right]=E_{XY}d\left(X,Y\right)+E_{YZ}d\left(Y,Z\right)\leq \Delta_{0}+\Delta_{1},$$

and then $P_{X}$ is a feasible PMF in Equation (A21). To prove the opposite inclusion, we observe that, for any $P_{Z}$ and $P_{X|Z}$ such that $D(P_{Z}∥P_{0})\leq \lambda$ and $E_{XZ}d\left(X,Z\right)\leq \Delta_{0}+\Delta_{1}$, it is possible to define a variable *Y*, and then two conditional PMFs $P_{Y|Z}$ and $P_{X|Y}$, such that $E_{XY}d\left(X,Y\right)\leq \Delta_{1}$ and $E_{YZ}d\left(Y,Z\right)\leq \Delta_{0}$. To do so, it is sufficient to let $P_{Y}$ be the convex combination of $P_{X}$ and $P_{Z}$, that is $P_{Y}=\alpha P_{X}+\left(1-\alpha \right)P_{Z}$ where $\alpha =\Delta_{0}/\left(\Delta_{0}+\Delta_{1}\right)$. With this choice for the marginal, we can define $P_{X|Y}$ so that $P_{XY}$ satisfies

(A23) $$\begin{array}{c} P_{XY}\left(i,j\right)=\left(1-\alpha \right)P_{XZ}\left(i,j\right)\;\forall i,\forall j\neq i,\\ P_{XY}\left(i,i\right)=\left(1-\alpha \right)P_{XZ}\left(i,i\right)+\alpha P_{X}\left(i\right)\;\forall i. \end{array}$$

By adopting the transportation theory perspective introduced towards the end of Section 6, we can look at $P_{X}$ and $P_{Y}$ as two ways of piling up a certain amount of soil; then, $P_{XY}$ can be interpreted as a map which moves $P_{X}$ into $P_{Y}$ ($P_{XY}\left(i,j\right)$ corresponds to the amount of soil moved from position *i* to *j*). The map in Equation (A23) is the one which leaves in place a percentage α of the mass and moves the remaining (1−α) percentage to fill the pile $\left(1-\alpha \right)P_{Z}$ according to map $\left(1-\alpha \right)P_{XZ}$.

Similarly, $P_{Y|Z}$ can be chosen such that $P_{YZ}$ satisfies

(A24) $$\begin{array}{c} P_{YZ}\left(i,j\right)=\alpha P_{XZ}\left(i,j\right)\;\forall i,\forall j\neq i,\\ P_{YZ}\left(i,i\right)=\alpha P_{XZ}\left(i,i\right)+\left(1-\alpha \right)P_{Z}\left(i\right)\;\forall i. \end{array}$$

It is easy to see that, with the above choices, $E_{XY}d\left(X,Y\right)=\left(1-\alpha \right)E_{XZ}d\left(X,Z\right)$ and $E_{YZ}d\left(Y,Z\right)=\alpha E_{XZ}d\left(X,Z\right)$. Then, $E_{XY}d\left(X,Y\right)\leq \left(1-\alpha \right)\left(\Delta_{0}+\Delta_{1}\right)\leq \Delta_{1}$ and $E_{YZ}d\left(Y,Z\right)\leq \Delta_{0}$. Consequently, any $P_{X}$ belonging to the set in Equation (A21) also belongs to the one in Equation (A20).

## Appendix B. Bayesian Detection Game

This appendix contains the proofs for Section 5.

### Appendix B.1. Proof of Theorem 5

Given the probability distributions $Q_{0}\left(y\right)$ and $Q_{1}\left(y\right)$ induced by $A_{\Delta_{0}}^{*}$ and $A_{\Delta_{1}}^{*}$, respectively, the optimal decision rule is deterministic and is given by the likelihood ratio test (LRT) [38]:

(A25) $$\frac{1}{n}\ln\frac{Q_{1}\left(y\right)}{Q_{0}\left(y\right)}\underset{H_{0}}{\overset{H_{1}}{≷}}a,$$

which proves the optimality of the decision rule in Equation (25).

To prove the asymptotic optimality of the decision rule in Equation (26), let us approximate $Q_{0}\left(y\right)$ and $Q_{1}\left(y\right)$ using the method of types as follows:

(A26) $$\begin{array}{ccc} Q_{0}\left(y\right) & = & \sum_{x}P_{0}\left(x\right)A_{\Delta_{0}}^{*}\left(y|x\right)\\ & \overset{\cdot }{=} & \sum_{x:\;d\left(x,y\right)\leq n\Delta_{0}}e^{-n[{\hat{H}}_{x}\left(X\right)+D\left({\hat{P}}_{x}∥P_{0}\right)]}\cdot e^{-n{\hat{H}}_{xy}\left(Y|X\right)}\\ & \overset{\cdot }{=} & \max_{x:\;d\left(x,y\right)\leq n\Delta_{0}}e^{n{\hat{H}}_{xy}\left(X|Y\right)}\cdot \left(e^{-n[{\hat{H}}_{x}\left(X\right)+D\left({\hat{P}}_{x}∥P_{0}\right)]}\right.\\ & & \;\left.\cdot e^{-n{\hat{H}}_{xy}\left(Y|X\right)}\right)\\ & = & \max_{x:\;d\left(x,y\right)\leq n\Delta_{0}}e^{-n[{\hat{H}}_{y}\left(Y\right)+D\left({\hat{P}}_{x}∥P_{0}\right)]}\\ & \overset{\left(a\right)}{=} & \exp\left\{-n\left[{\hat{H}}_{y}\left(Y\right)+\right.\right.\\ & & \left.\left.\;+\min_{\left\{{\hat{P}}_{x|y}:E_{xy}d\left(X,Y\right)\leq \Delta_{0}\right\}}D\left({\hat{P}}_{x}∥P_{0}\right)\right]\right\}\\ & = & \exp\left\{-n[{\hat{H}}_{y}\left(Y\right)+{\tilde{D}}_{\Delta_{0}}^{n}\left({\hat{P}}_{y},P_{0}\right)]\right\}, \end{array}$$

where in (a) we exploited the additivity of the distortion function *d*. Similarly,

(A27) $$Q_{1}\left(y\right)\overset{\cdot }{=}\exp\left\{-n[{\hat{H}}_{y}\left(Y\right)+{\tilde{D}}_{\Delta_{1}}^{n}\left({\hat{P}}_{y},P_{1}\right)]\right\}.$$

Thus, we have the following asymptotic approximation to the LRT:

(A28) $${\tilde{D}}_{\Delta_{0}}^{n}\left({\hat{P}}_{y},P_{0}\right)-{\tilde{D}}_{\Delta_{1}}^{n}\left({\hat{P}}_{y},P_{1}\right)\underset{H_{0}}{\overset{H_{1}}{≷}}a,$$

which proves the second part of the theorem.

### Appendix B.2. Proof of Theorem 7

To make the expression of $u(P_{\mathrm{FN}}\left(\Phi^{†},\left(A_{\Delta_{0}}^{*},A_{\Delta_{1}}^{*}\right)\right))$ explicit, let us first evaluate the two error probabilities at equilibrium. Below, we derive the lower and upper bound on the probability of ***y*** under $H_{1}$, when the attack channel is $A_{\Delta_{1}}^{*}$:

(A29) $${\left(n+1\right)}^{-|A∥A-1|}e^{-n[{\hat{H}}_{y}\left(Y\right)+{\tilde{D}}_{\Delta_{1}}^{n}\left({\hat{P}}_{y},P_{1}\right)]}\leq Q_{1}^{*}\left(y\right)<{\left(n+1\right)}^{{\left|A\right|}^{2}}e^{-n[{\hat{H}}_{y}\left(Y\right)+{\tilde{D}}_{\Delta_{1}}^{n}\left({\hat{P}}_{y},P_{1}\right)]}.$$

The same bounds hold for $Q_{0}^{*}\left(y\right)$, with ${\tilde{D}}_{\Delta_{0}}$ replacing ${\tilde{D}}_{\Delta_{0}}$. For the FN probability, the upper bound is

(A30) $$\begin{array}{ccc} P_{\mathrm{FN}}\left(\Phi^{†},A_{\Delta_{1}}^{*}\right) & = & \sum_{y}Q_{1}^{*}\left(y\right)\cdot \Phi^{†}\left(H_{0}|y\right)\\ & = & \sum_{y:{\tilde{D}}_{\Delta_{0}}^{n}\left({\hat{P}}_{y},P_{0}\right)-{\tilde{D}}_{\Delta_{1}}^{n}\left({\hat{P}}_{y},P_{1}\right)<a}Q_{1}^{*}\left(y\right)\\ & \leq & {\left(n+1\right)}^{{\left|A\right|}^{2}}\sum_{y:{\tilde{D}}_{\Delta_{0}}^{n}\left({\hat{P}}_{y},P_{0}\right)-{\tilde{D}}_{\Delta_{1}}^{n}\left({\hat{P}}_{y},P_{1}\right)<a}e^{-n[{\hat{H}}_{y}+{\tilde{D}}_{\Delta_{1}}^{n}\left({\hat{P}}_{y},P_{1}\right)]}\\ & \leq & {\left(n+1\right)}^{{\left|A\right|}^{2}+\left|A\right|}\max_{{\hat{P}}_{y}:{\tilde{D}}_{\Delta_{0}}^{n}\left({\hat{P}}_{y},P_{0}\right)-{\tilde{D}}_{\Delta_{1}}^{n}\left({\hat{P}}_{y},P_{1}\right)<a}e^{-n{\tilde{D}}_{\Delta_{1}}^{n}\left({\hat{P}}_{y},P_{1}\right)}\\ & = & {\left(n+1\right)}^{{\left|A\right|}^{2}+\left|A\right|}\exp\left\{-n\left(\min_{{\hat{P}}_{y}:{\tilde{D}}_{\Delta_{0}}^{n}\left({\hat{P}}_{y},P_{0}\right)-{\tilde{D}}_{\Delta_{1}}^{n}\left({\hat{P}}_{y},P_{1}\right)<a}{\tilde{D}}_{\Delta_{1}}^{n}\left({\hat{P}}_{y},P_{1}\right)\right)\right\}. \end{array}$$

Then,

(A31) $$\begin{array}{c} -\underset{n\to \infty }{lim sup}\frac{1}{n}\ln\left(P_{\mathrm{FN}}\left(\Phi^{†},A_{\Delta_{1}}^{*}\right)\right)\leq \min_{P_{Y}:{\tilde{D}}_{\Delta_{0}}\left(P_{Y},P_{0}\right)-{\tilde{D}}_{\Delta_{1}}\left(P_{Y},P_{1}\right)\leq a}{\tilde{D}}_{\Delta_{1}}\left(P_{Y},P_{1}\right). \end{array}$$

For the lower bound,

(A32) $$\begin{array}{ccc} P_{\mathrm{FN}}\left(\Phi^{†},A_{\Delta_{1}}^{*}\right) & \geq & {\left(n+1\right)}^{-|A∥A-1|}\sum_{y:{\tilde{D}}_{\Delta_{0}}^{n}\left({\hat{P}}_{y},P_{0}\right)-{\tilde{D}}_{\Delta_{1}}^{n}\left({\hat{P}}_{y},P_{1}\right)<a}e^{-n[{\hat{H}}_{y}+{\tilde{D}}_{\Delta_{1}}^{n}\left({\hat{P}}_{y},P_{1}\right)]}\\ & \geq & {\left(n+1\right)}^{-|A∥A-1|}\max_{{\hat{P}}_{y}:{\tilde{D}}_{\Delta_{0}}^{n}\left({\hat{P}}_{y},P_{0}\right)-{\tilde{D}}_{\Delta_{1}}^{n}\left({\hat{P}}_{y},P_{1}\right)<a}e^{-n{\tilde{D}}_{\Delta_{1}}^{n}\left({\hat{P}}_{y},P_{1}\right)}\\ & = & {\left(n+1\right)}^{-|A∥A-1|}\exp\left\{-n\left(\min_{{\hat{P}}_{y}:{\tilde{D}}_{\Delta_{0}}^{n}\left({\hat{P}}_{y},P_{0}\right)-{\tilde{D}}_{\Delta_{1}}^{n}\left({\hat{P}}_{y},P_{1}\right)<a}{\tilde{D}}_{\Delta_{1}}^{n}\left({\hat{P}}_{y},P_{1}\right)\right)\right\}. \end{array}$$

Then,

(A33) $$\begin{array}{ccc} -\underset{n\to \infty }{lim inf}\frac{1}{n}\ln\left(P_{\mathrm{FN}}\left(\Phi^{†},A_{\Delta_{1}}^{*}\right)\right) & \geq & \lim_{n\to \infty }{\tilde{D}}_{\Delta_{1}}^{n}\left({\hat{P}}_{y},P_{1}\right)\\ & = & \min_{P_{Y}:{\tilde{D}}_{\Delta_{0}}\left(P_{Y},P_{0}\right)-{\tilde{D}}_{\Delta_{1}}\left(P_{Y},P_{1}\right)\leq a}{\tilde{D}}_{\Delta_{1}}\left(P_{Y},P_{1}\right), \end{array}$$

where ${\hat{P}}_{y}$ is a properly chosen PMF, belonging to the set $\left\{{\tilde{D}}_{\Delta_{0}}^{n}\left({\hat{P}}_{y},P_{0}\right)-{\tilde{D}}_{\Delta_{1}}^{n}\left({\hat{P}}_{y},P_{1}\right)<a\right\}$ for every *n*, and such that ${\hat{P}}_{y}\to P_{Y}^{*}$ where

(A34) $$P_{Y}^{*}=\arg\min_{P_{Y}:{\tilde{D}}_{\Delta_{0}}\left(P_{Y},P_{0}\right)-{\tilde{D}}_{\Delta_{1}}\left(P_{Y},P_{1}\right)\leq a}{\tilde{D}}_{\Delta_{1}}\left(P_{Y},P_{1}\right).$$

By Property 1, set $\left\{{\tilde{D}}_{\Delta_{0}}^{n}\left({\hat{P}}_{y},P_{0}\right)-{\tilde{D}}_{\Delta_{1}}^{n}\left({\hat{P}}_{y},P_{1}\right)<a\right\}$ is dense in $\left\{P_{Y}:{\tilde{D}}_{\Delta_{0}}\left(P_{Y},P_{0}\right)-{\tilde{D}}_{\Delta_{1}}\left(P_{Y},P_{1}\right)\leq a\right\}$ and then such a sequence of PMFs can always be found.

By combining Equations (A31) and (A33), we get

(A35) $$\begin{array}{c} \varepsilon_{\mathrm{FN}}=-\lim_{n\to \infty }\frac{1}{n}\ln\left(P_{\mathrm{FN}}\left(\Phi^{†},A_{\Delta_{1}}^{*}\right)\right)=\min_{P_{Y}:{\tilde{D}}_{\Delta_{0}}\left(P_{Y},P_{0}\right)-{\tilde{D}}_{\Delta_{1}}\left(P_{Y},P_{1}\right)\leq a}{\tilde{D}}_{\Delta_{1}}\left(P_{Y},P_{1}\right). \end{array}$$

Therefore, from Equations (A30) and (A32) we have

(A36) $$\begin{array}{c} P_{\mathrm{FN}}\left(\Phi^{†},A_{\Delta_{1}}^{*}\right)≐\exp\left\{-n\left(\min_{{\hat{P}}_{y}:{\tilde{D}}_{\Delta_{0}}^{n}\left({\hat{P}}_{y},P_{0}\right)-{\tilde{D}}_{\Delta_{1}}^{n}\left({\hat{P}}_{y},P_{1}\right)<a}{\tilde{D}}_{\Delta_{1}}^{n}\left({\hat{P}}_{y},P_{1}\right)\right)\right\}, \end{array}$$

and the limit of $\frac{1}{n}\ln P_{\mathrm{FN}}$ exists and is finite.

Similar bounds can be derived for the FP probability, resulting in

(A37) $$\begin{array}{c} P_{\mathrm{FP}}\left(\Phi^{*},A_{\Delta_{0}}^{*}\right)≐\exp\left\{-n\left(\min_{{\hat{P}}_{y}:{\tilde{D}}_{\Delta_{0}}^{n}\left({\hat{P}}_{y},P_{0}\right)-{\tilde{D}}_{\Delta_{1}}^{n}\left({\hat{P}}_{y},P_{1}\right)\geq a}{\tilde{D}}_{\Delta_{0}}^{n}\left({\hat{P}}_{y},P_{0}\right)\right)\right\}, \end{array}$$

and in particular

(A38) $$\begin{array}{c} \varepsilon_{\mathrm{FP}}=-\lim_{n\to \infty }\frac{1}{n}\ln\left(P_{\mathrm{FP}}\left(\Phi^{*},A_{\Delta_{0}}^{*}\right)\right)=\min_{P_{Y}:{\tilde{D}}_{\Delta_{0}}\left(P_{Y},P_{0}\right)-{\tilde{D}}_{\Delta_{1}}\left(P_{Y},P_{1}\right)\geq a}{\tilde{D}}_{\Delta_{0}}\left(P_{Y},P_{0}\right). \end{array}$$

From Equation (A38), we see that, as argued, the profile $\left(\Phi^{†},\left(A_{\Delta_{0}}^{*},A_{\Delta_{1}}^{*}\right)\right)$ leads to a FP exponent always at least as large as *a*.

We are now ready to evaluate the asymptotic behavior of the payoff of the Bayesian detection game:

(A39) $$\begin{array}{ccc} u & = & P_{\mathrm{FN}}\left(\Phi^{†},A_{\Delta_{1}}^{*}\right)+e^{an}P_{\mathrm{FP}}\left(\Phi^{†},A_{\Delta_{0}}^{*}\right)\\ & ≐ & \max\{P_{\mathrm{FN}}\left(\Phi^{†},A_{\Delta_{1}}^{*}\right),e^{an}P_{\mathrm{FP}}\left(\Phi^{†},A_{\Delta_{0}}^{*}\right)\}\\ & ≐ & \exp\left\{-n\min\left(\min_{{\hat{P}}_{y}:{\tilde{D}}_{\Delta_{0}}^{n}\left({\hat{P}}_{y},P_{0}\right)-{\tilde{D}}_{\Delta_{1}}^{n}\left({\hat{P}}_{y},P_{1}\right)<a}{\tilde{D}}_{\Delta_{1}}^{n}\left({\hat{P}}_{y},P_{1}\right),\min_{{\hat{P}}_{y}:{\tilde{D}}_{\Delta_{0}}^{n}\left({\hat{P}}_{y},P_{0}\right)-{\tilde{D}}_{\Delta_{1}}^{n}\left({\hat{P}}_{y},P_{1}\right)\geq a}\left({\tilde{D}}_{\Delta_{0}}^{n}\left({\hat{P}}_{y},P_{0}\right)-a\right)\right)\right\}\\ & = & \exp\left\{-n\min_{P_{y}}\left(\max\left\{{\tilde{D}}_{\Delta_{1}}^{n}\left({\hat{P}}_{y},P_{1}\right),\left({\tilde{D}}_{\Delta_{0}}^{n}\left({\hat{P}}_{y},P_{0}\right)-a\right)\right\}\right)\right\}\\ & ≐ & \exp\left\{-n\min_{P_{Y}}\left(\max\left\{{\tilde{D}}_{\Delta_{1}}\left(P_{Y},P_{1}\right),\left({\tilde{D}}_{\Delta_{0}}\left(P_{Y},P_{0}\right)-a\right)\right\}\right)\right\}, \end{array}$$

where the asymptotic equality in the last line follows from the density of the set of empirical probability distributions of *n*-length sequences in the probability simplex and from the continuity of the to-be-minimized expression in round brackets as a function of $P_{Y}$.

## Appendix C. Source Distinguishability

This appendix contains the proofs for Section 6.

### Appendix C.1. Proof of Theorem 9

The theorem directly follows from Theorem 7. In fact, by letting

(A40) $$e_{a}\left(P_{Y}\right)=\max\left\{{\tilde{D}}_{\Delta_{1}}\left(P_{Y},P_{1}\right),{\tilde{D}}_{\Delta_{0}}\left(P_{Y},P_{0}\right)-a\right\},$$

a≥0, the limit in Equation (29) can be derived as follows:

(A41) $$\begin{array}{cc} \lim_{a\to 0}\;\min_{P_{Y}}e_{a}\left(P_{Y}\right) & =\min_{P_{Y}}\;\lim_{a\to 0}e_{a}\left(P_{Y}\right)\\ & =\min_{P_{Y}}\left(\max\left\{{\tilde{D}}_{\Delta_{1}}\left(P_{Y},P_{1}\right),{\tilde{D}}_{\Delta_{0}}\left(P_{Y},P_{0}\right)\right\}\right), \end{array}$$

where the order of limit and minimum can be exchanged because of the uniform convergence of $e_{a}\left(P_{Y}\right)$ to $e_{0}\left(P_{Y}\right)$ as *a* tends to 0.

### Appendix C.2. Proof of Corollary 2

The corollary can be proven by exploiting the fact that, when *d* is a metric, the *EMD* is a metric and then ${EMD}_{d}\left(P_{0},P\right)$ satisfies the triangular inequality. In this case, it is easy to argue that the $P_{Y}$ achieving the minimum in Equation (32) is the one for which the triangular relation holds at the equality, which corresponds to the convex combination of $P_{0}$ and *P* (i.e., the PMF lying on the straight line between $P_{0}$ and *P*) with combination coefficient α such that ${EMD}_{d}\left(P_{0},P_{Y}\right)$ (or equivalently, by symmetry, ${EMD}_{d}\left(P_{Y},P_{0}\right)$) is exactly equal to $\Delta_{0}$.

Formally, let $X∼P_{0}$ and Z∼P. We want to find the PMF $P_{Y}$ which solves

(A42) $$\min_{P_{Y}:{EMD}_{d}\left(P_{Y},P_{0}\right)\leq \Delta_{0}}{EMD}_{d}\left(P_{Y},P\right).$$

For any $Y∼P_{Y}$ and any choice of $P_{XY}$ and $P_{YZ}$ (that is, $P_{Y|X}$ and $P_{Z|Y}$), by exploiting the triangular inequality property of the distance, we can write

(A43) $$\begin{array}{c} E_{XZ}d\left(X,Z\right)\leq E_{XY}d\left(X,Y\right)+E_{YZ}d\left(Y,Z\right), \end{array}$$

where $P_{XZ}$ can be any joint distribution with marginals $P_{0}$ and *P*. Then,

(A44) $$EMD\left(P_{0},P\right)\leq E_{XY}d\left(X,Y\right)+E_{YZ}d\left(Y,Z\right).$$

From the arbitrariness of the choice of $P_{XY}$ and $P_{YZ}$, if we let $P_{XY}^{*}$ and $P_{YZ}^{*}$ be the joint distributions achieving the *EMD* between *X* and *Y*, and *Y* and *Z*, we get

(A45) $$EMD\left(P_{0},P\right)\leq EMD\left(P_{0},P_{Y}\right)+EMD\left(P_{Y},P\right).$$

From the above relation, we can derive the following lower bound for the to-be-minimized quantity in Equation (A42):

(A46)EMD(PY,P)≥EMD(P0,P)−EMD(P0,PY)(A47)≥EMD(P0,P)−Δ0.

We now show that $P_{Y}$ defined as in Equation (33) achieves the above lower bound while satisfying the constraint $EMD\left(P_{0},P_{Y}\right)\leq \Delta_{0}$, and then get the minimum value in Equation (A42).

Let $P_{XZ}^{*}$ be the joint distribution achieving the *EMD* between *X* and *Z*. Then, $E_{XZ}^{*}d\left(X,Z\right)=EMD\left(P_{0},P\right)$ (where the star on the apex indicates that the expectation is taken under $P_{XZ}^{*}$). Given the marginal $P_{Y}=\alpha P_{0}+\left(1-\alpha \right)P$, we can define $P_{XY}$ and $P_{YZ}$, starting from $P_{XZ}^{*}$, as in the proof of Theorem 4 (Equations (A23) and (A24)). With this choice, $E_{XY}d\left(X,Y\right)=\left(1-\alpha \right)EMD\left(P_{0},P\right)$ and $E_{YZ}d\left(Y,Z\right)=\alpha EMD\left(P_{0},P\right)$. Then, for the value of α in Equation (33) we have that $E_{XY}d\left(X,Y\right)=\Delta_{0}$ and

(A48) $$E_{YZ}d\left(Y,Z\right)=EMD\left(P_{0},P\right)-\Delta_{0}.$$

By combining Equations (A47) and (A48), we argue that $EMD\left(P_{Y},P\right)=EMD\left(P_{0},P\right)-\Delta_{0}$ (we also argue that the choice made for $P_{YZ}$ minimizes the expected distortion between *Y* and *Z*, i.e., it yields $E_{YZ}d\left(Y,Z\right)=EMD\left(P_{Y},P\right)$. Furthermore, being $E_{XY}d\left(X,Y\right)=\Delta_{0}$, it holds $EMD\left(P_{Y},P\right)=EMD\left(P_{0},P\right)-E_{XY}d\left(X,Y\right)$ and then, from the triangular inequality in Equation (A45), it follows that $EMD\left(P_{0},P_{Y}\right)=E_{XY}d\left(X,Y\right)=\Delta_{0}$) . Therefore, $P_{Y}$ in Equation (33) solves Equation (A42).

To prove the second part of the corollary, we just need to observe that a PMF *P* belongs to the indistinguishability set in Equation (32) if and only if

(A49) $$EMD\left(P_{Y},P\right)=EMD\left(P_{0},P\right)-\Delta_{0}\leq \Delta_{1},$$

that is $EMD\left(P_{0},P\right)\leq \Delta_{0}+\Delta_{1}$.

From the above proof, we notice that, for any *P* in the set in Equation (34), i.e., such that ${EMD}_{d}\left(P_{0},P\right)\leq \Delta_{0}+\Delta_{1}$, the PMF $P_{Y}=\alpha P_{0}+\left(1-\alpha \right)P$ with α as in Equation (33) satisfies *EMD*$\left(P_{Y},P_{0}\right)=\Delta_{0}$ and *EMD*$\left(P_{Y},P_{1}\right)=\Delta_{1}$ for any choice of *d*. Then, when *d* is not a metric, the region in Equation (34) is contained in the indistinguishability region.

### Appendix C.3. Proof of Corollary 3

By inspecting the minimization in Equation (32), we see that, for any source *P* that cannot be distinguished from $P_{0}$, it is possible to find a source $P_{Y}$ such that ${EMD}_{d}\left(P_{Y},P\right)\leq \Delta_{1}$ and ${EMD}_{d}\left(P_{Y},P_{0}\right)\leq \Delta_{0}$. To prove the corollary, we need to show that such *P* lies inside the set defined in Equation (35).

We give the following definition. Given two random variables *X* and *Y*, the Hölder inequality applied to the expectation function [36] reads:

(A50) $$E_{XY}\left|XY\right|\leq {\left(E_{X}{\left[|X\right|}^{r}]\right)}^{1/r}{\left(E_{Y}{\left[|Y\right|}^{q}]\right)}^{1/q},$$

where r≥1 and q=r/(r−1), namely, the Hölder conjugate of *r*.

We use the notation $E_{XY}^{*}$ for the expectation of the pair (X,Y) when the probability map is the one achieving the $EMD(P_{X},P_{Y})$, namely $P_{XZ}^{*}$. Then, we can write:

(A51) $$\begin{array}{cc} {EMD}_{L_{p}^{p}}\left(P_{0},P\right) & =E_{XZ}^{*}[||X-{Z||}^{p}]\\ & \overset{\left(a\right)}{\leq }E_{XYZ}^{*}[(||X-Y||+||Y-{Z||)}^{p}]\\ & \overset{\left(b\right)}{\leq }E_{XYZ}\left[||X-{Y||}^{p}+||Y-{Z||}^{p}+p\cdot ||X-{Y||}^{p-1}\;||Y-Z||+\right.\\ & \left.\;+p\left(p-1\right)/2\cdot ||X-{Y||}^{p-2}\;||Y-{Z||}^{2}+\ldots \ldots +p\cdot ||X-Y||\;||Y-{Z||}^{p-1}\right]\\ & =E_{XYZ}[||X-{Y||}^{p}]+E_{XYZ}[||Y-{Z||}^{p}]+p\cdot E_{XYZ}[||X-{Y||}^{p-1}\;||Y-Z||]+\\ & \;+p\left(p-1\right)/2\cdot E_{XYZ}[||X-{Y||}^{p-2}\;||Y-{Z||}^{2}]+\ldots .\\ & \;\ldots \ldots +p\cdot E_{XYZ}[||X-Y||\;||Y-{Z||}^{p-1}]\\ & \overset{\left(c\right)}{\leq }E_{XYZ}[||X-{Y||}^{p}]+E_{XYZ}[||Y-{Z||}^{p}]+p\cdot {(E_{XYZ}[||X-{Y||}^{p}])}^{\frac{p-1}{p}}(E_{XYZ}[||Y-{Z||}^{p}{])}^{\frac{1}{p}}\\ & \;+p\left(p-1\right)/2\cdot (E_{XYZ}[||X-{Y||}^{p}{)}^{\frac{p-2}{p}}(E_{XYZ}[||Y-{Z||}^{p}{])}^{\frac{2}{p}}+\ldots \\ & \;\ldots +p\cdot (E_{XYZ}[||X-{Y||}^{p}{])}^{\frac{1}{p}}(E_{XYZ}[||Y-{Z||}^{p}{])}^{\frac{p-1}{p}}\\ & =E_{XY}[||X-{Y||}^{p}]+E_{YZ}[||Y-{Z||}^{p}]+p\cdot {(E_{XY}[||X-{Y||}^{p}])}^{\frac{p-1}{p}}(E_{YZ}[||Y-{Z||}^{p}{])}^{\frac{1}{p}}\\ & \;+p\left(p-1\right)/2\cdot (E_{XY}[||X-{Y||}^{p}{])}^{\frac{p-2}{p}}(E_{YZ}[||Y-{Z||}^{p}{])}^{\frac{2}{p}}+\ldots \\ & \;\ldots +p\cdot (E_{XY}[||X-{Y||}^{p}{])}^{\frac{1}{p}}(E_{YZ}[||Y-{Z||}^{p}{])}^{\frac{p-1}{p}}\\ & ={\left((E_{XYZ}[||X-{Y||}^{p}{])}^{1/p}+(E_{XYZ}[||Y-{Z||}^{p}{])}^{1/p}\right)}^{p}\\ & \leq {\left(\Delta_{0}^{1/p}+\Delta_{1}^{1/p}\right)}^{p}, \end{array}$$

where in (a) we considered the joint distribution $P_{XYZ}$ such that $\sum_{Z}P_{XYZ}=P_{XY}^{*}$, $\sum_{X}P_{XYZ}=P_{YZ}^{*}$ (and, consequently, $\sum_{Y}P_{XYZ}=P_{XZ}^{*}$) and in (b) we developed the *p*-power of the binomial (binomial theorem). Finally, in (c), we applied the Hölder’s inequality to the various terms of Newton’s binomial: specifically, for each term $E_{XYZ}[||X-{Y||}^{p-t}\;||Y-{Z||}^{t}]$, with t=1,…,p−1, the Hölder inequality is applied with r=p/(p−t) (and q=r/(r−1)).
